# Directed Evolution of T7 RNA Polymerase Minimizes dsRNA By-product and Enables High-Fidelity mRNA Synthesis for Demanding Therapeutic Applications

**DOI:** 10.34133/research.1172

**Published:** 2026-02-25

**Authors:** Weitong Qin, Ting Nie, Mohan Hei, Liang Li, Yunjie Pan, Manjie Luo, Guang-Yu Yang

**Affiliations:** ^1^ State Key Laboratory of Microbial Metabolism, Joint International Research Laboratory of Metabolic and Developmental Sciences, School of Life Sciences and Biotechnology, Shanghai Jiao Tong University, Shanghai 200240, China.; ^2^ Hzymes Biotechnology Co., Ltd., Wuhan, Hubei 430010, China.; ^3^ Institute of Key Biological Raw Material, Shanghai Academy of Experimental Medicine, Shanghai 201401, China.

## Abstract

T7 RNA polymerase (T7 RNAP) is the most widely used enzyme for synthesizing therapeutic mRNA. However, RNA transcribed by T7 RNAP often contains double-stranded RNA (dsRNA) by-products that trigger innate immune responses and complicate purification. Here, we report an engineered T7 RNAP variant, M30, which exhibits higher catalytic efficiency and reduced dsRNA by-product formation. M30 was developed through 4 rounds of directed evolution using an ultrahigh-throughput aptamer-based fluorescence-activated droplet sorting system. M30 displays a 10-fold increase in catalytic efficiency over wild-type T7 RNAP at 37 °C, along with markedly enhanced thermostability and approximately 10-fold lower production of dsRNA by-products. mRNAs synthesized with M30 achieve efficient protein expression in human cells and in mice, while eliciting reduced immunogenicity compared with mRNAs produced by wild-type T7 RNAP. Biophysical assays and structural analyses suggest that these improvements result from increased DNA template binding affinity and decreased RNA binding affinity. Together, these features make M30 a promising catalyst for high-quality therapeutic mRNA production.

## Introduction

mRNA technology is regarded as a next-generation therapeutic platform owing to its rapid manufacturing, design flexibility, and cost-effective production [1–4]. Currently, large-scale mRNA synthesis relies mainly on in vitro transcription (IVT) using T7 RNA polymerase (T7 RNAP). However, IVT often generates undesired double-stranded RNA (dsRNA), which can cause side effects such as reduced expression, cytotoxic inflammation, and even cell death [5]. Following the COVID-19 pandemic, the focus of mRNA therapeutics has shifted from preventive vaccines to therapeutic applications, as the demonstrated efficacy, scalability, and modularity of mRNA platforms have revealed new opportunities in cancer vaccines, protein replacement therapies, and in vivo chimeric antigen receptor T-cell (CAR-T) therapies [6,7]. These modalities typically require mRNA doses 100 to 1,000 times higher than those used for preventive vaccines, making stringent control of impurities critical for drug safety [8–10].

Removing dsRNA from mRNA products is challenging owing to their similar size and physicochemical properties. Current strategies largely rely on costly and time-consuming post-transcriptional chromatographic purification [11]. Various approaches have been explored to minimize dsRNA formation by optimizing reaction conditions, including using modified nucleotides [12], reducing Mg^2+^ concentration [13], adding competing 3′-capture oligonucleotides [14], incorporating urea [15], increasing reaction temperature, or employing high-affinity promoters under high-salt conditions [16]. However, these strategies can compromise RNA yield and may introduce additional contaminants.

Recently, several studies have focused on engineering T7 RNAP to reduce dsRNA by-product formation. Engineered variants such as G47A+884G and G47W, which produce lower levels of dsRNA, were developed using structure-guided rational design and phage-assisted continuous evolution [17]. Another study reported a commercially available thermostable variant, NEB-Hi-T7, which markedly reduces dsRNA formation at transcription temperatures above 48.5 °C. Despite progress, existing engineering variants such as NEB-Hi-T7 are limited by inconsistent yields. Thus, the industry continues to seek T7 RNAP variants that combine superior transcription activity, enhanced stability, and minimal dsRNA by-products for demanding therapeutic applications [6].

**Fig. 1.**
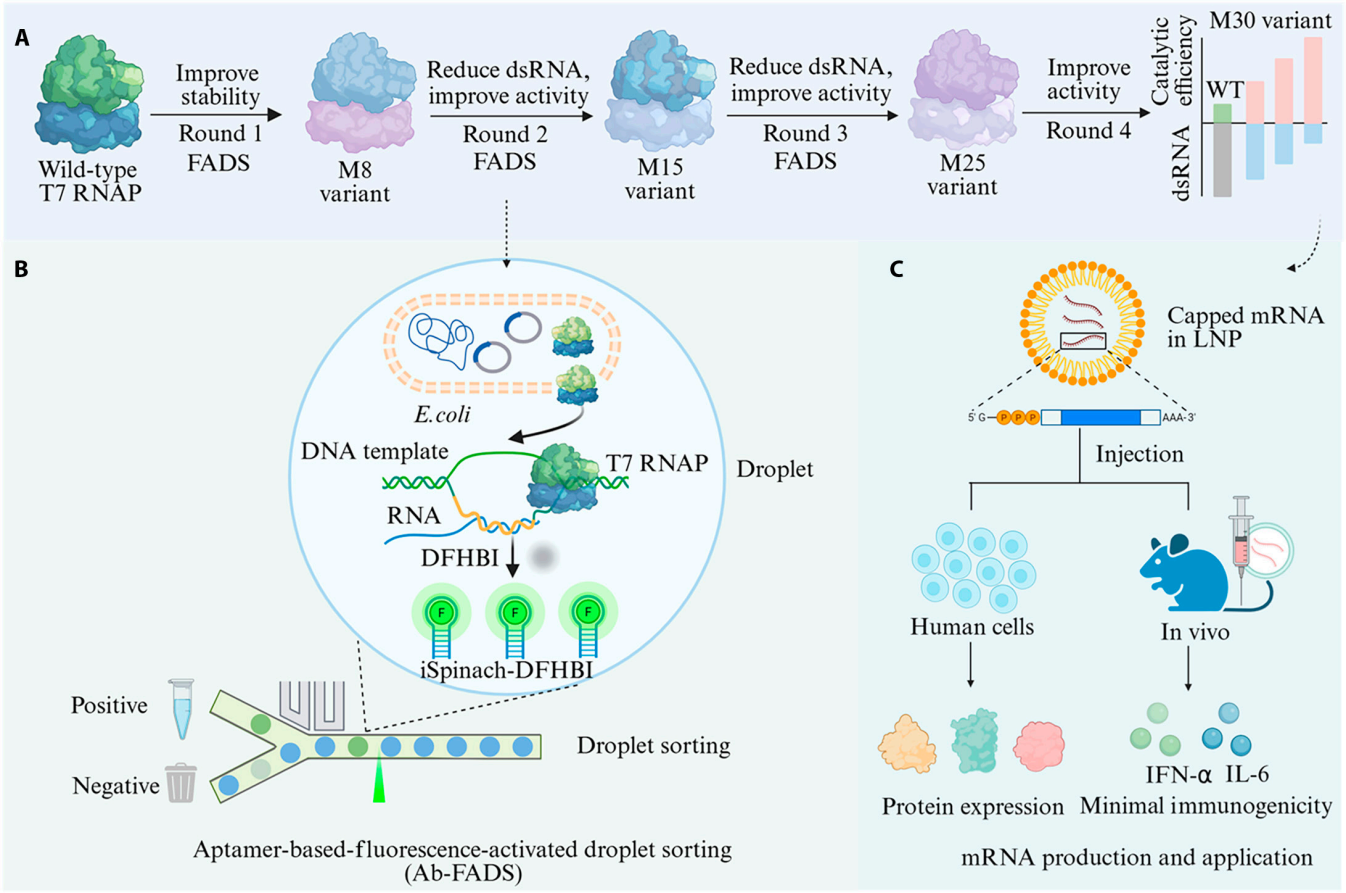
Directed evolution of T7 RNAP using an aptamer-based FADS platform. (A) A T7 RNAP variant with reduced dsRNA by-products and enhanced catalytic efficiency was obtained through 4 rounds of evolution. (B) Principle of the Ab-FADS platform. A single *E. coli* cell expressing a T7 RNAP variant and the T-STAR detection system is co-encapsulated in a water-in-oil droplet. The DNA template contains a T7 promoter and a tandem iSpinach RNA aptamer. During in vitro transcription, only full-length transcripts bind DFHBI, producing a fluorescent signal directly proportional to enzyme activity. (C) Different types of mRNAs are synthesized using the M30 variant and either transfected into human cells or injected into mice to evaluate protein expression levels and immunogenicity.

Here, we performed directed evolution of T7 RNAP using an ultrahigh-throughput fluorescence-activated droplet sorting (FADS) platform capable of screening up to 10^8^ variants per day (Fig. 1). Four rounds of semirational design, targeting the solvent-inaccessible cavity of T7 RNAP—a region potentially involved in dsRNA formation—produced the M30 variant, which exhibits reduced dsRNA and 3′-heterogeneity by-products, along with enhanced catalytic activity. Biophysical assays and molecular dynamics (MD) simulations were also employed to investigate the mechanisms underlying these functional improvements.

## Results

### Developing an aptamer-based FADS system to screen T7 RNAP libraries

The first step in developing an improved T7 RNAP variant was establishing a fluorescence-coupling strategy to screen large mutant libraries. Briefly, we designed a double-stranded DNA template containing a T7 promoter sequence followed by the iSpinach RNA aptamer, based on the previously reported STAR system, which enables real-time monitoring of RNA synthesis (Fig. S1A) [18–20]. To enhance the signal-to-noise ratio, we employed a quadrupled iSpinach aptamer, which showed a strong linear correlation with T7 RNAP concentrations ranging from 0.4 to 5 nM (Fig. S3). Because only the fully folded iSpinach aptamer binds DFHBI, fluorescence is emitted exclusively from full-length transcription products. Notably, our aptamer-based fluorescence-activated droplet sorting (Ab-FADS) system also performed robustly with crude enzyme preparations from *Escherichia coli* cell lysates (Figs. S3D and S4A).

The complete workflow for Ab-FADS-based screening of T7 RNAP variants is illustrated in Fig. S1. The system demonstrates excellent sensitivity for detecting T7 RNAP activity at the single-cell level within microdroplets (Fig. S4B). The Ab-FADS is compatible with nonstandard reaction conditions, such as high temperatures, and with modified nucleotides, including pseudouridine (Fig. S5), offering greater flexibility than in vivo screening methods such as phage-assisted continuous evolution. The sorting throughput reaches up to 10^8^ clones per day, enabling the rapid identification of rare beneficial variants and markedly shortening the directed evolution cycle for T7 RNAP. A pilot screen separating droplets containing *E*. *coli* cells expressing wild-type (WT) T7 RNAP from those with an inactive T7 RNAP mutant achieved 99.4% sorting accuracy after a single round, demonstrating that the Ab-FADS system can efficiently and reliably screen T7 RNAP activities (Fig. S6).

### Directed evolution of T7 RNAP variants for enhanced catalytic activity at increased temperatures

Previous studies have shown that dsRNA by-products during T7 RNAP-catalyzed transcription are partly caused by self-primed extension of nascent mRNA [21]. Based on reports that high temperatures can reduce dsRNA formation [17], we initially aimed to enhance T7 RNAP performance by conducting the first round of directed evolution screening at increased temperatures. Using error-prone polymerase chain reaction (PCR), we generated a random mutagenesis library of approximately 2 million mutants in *E*. *coli* BL(DE3) cells, with an average of 2 to 3 mutations per clone. Prior to screening, droplets were pretreated at 45 °C for 15 min to impose selection pressure for both improved thermostability and catalytic activity. We then sorted the top 0.18% of positive droplets at 500 Hz for 7 h, screening roughly 1.4 × 10^7^ cells.

We then performed a focused follow-up analysis of high-ranking candidates using 96-well plates after 2 successive rounds of enrichment (Fig. S7). Crude enzyme solutions from these positive clones were pretreated at 45 °C for 15 min, and their relative catalytic activity at 37 °C was measured. Three improved variants, namely, S397A, S397W, and S397W/S430P, were identified in the first round (Table S1). The best purified variant, S397W/S430P, exhibited 5.6-fold higher catalytic activity than the WT following heat pretreatment and was selected for the next round of evolution.

### Evolution of T7 RNAP variants to reduce dsRNA by-products and enhance activity

A seminal study demonstrated that solvent-inaccessible cavities can serve as allosteric nodes, modulating enzyme function through conformational and energetic coupling [22]. We hypothesized that mutation-tolerant internal residues in T7 RNAP could indirectly influence RNA release and/or reannealing, thereby affecting dsRNA formation. Indeed, a previous study showed that filling a C-terminal Phe-Ala-Phe-Ala^883^ solvent-inaccessible cavity with an additional glycine (Gly884) reduced dsRNA formation, although it also decreased catalytic efficiency due to its proximity to the active site [23]. Based on this, we designed a focused mutagenesis library targeting regions away from the catalytic site. To assess changes within the internal cavity, we incorporated energy calculations, as cavity size is known to influence enzyme stability [23].

**Fig. 2.**
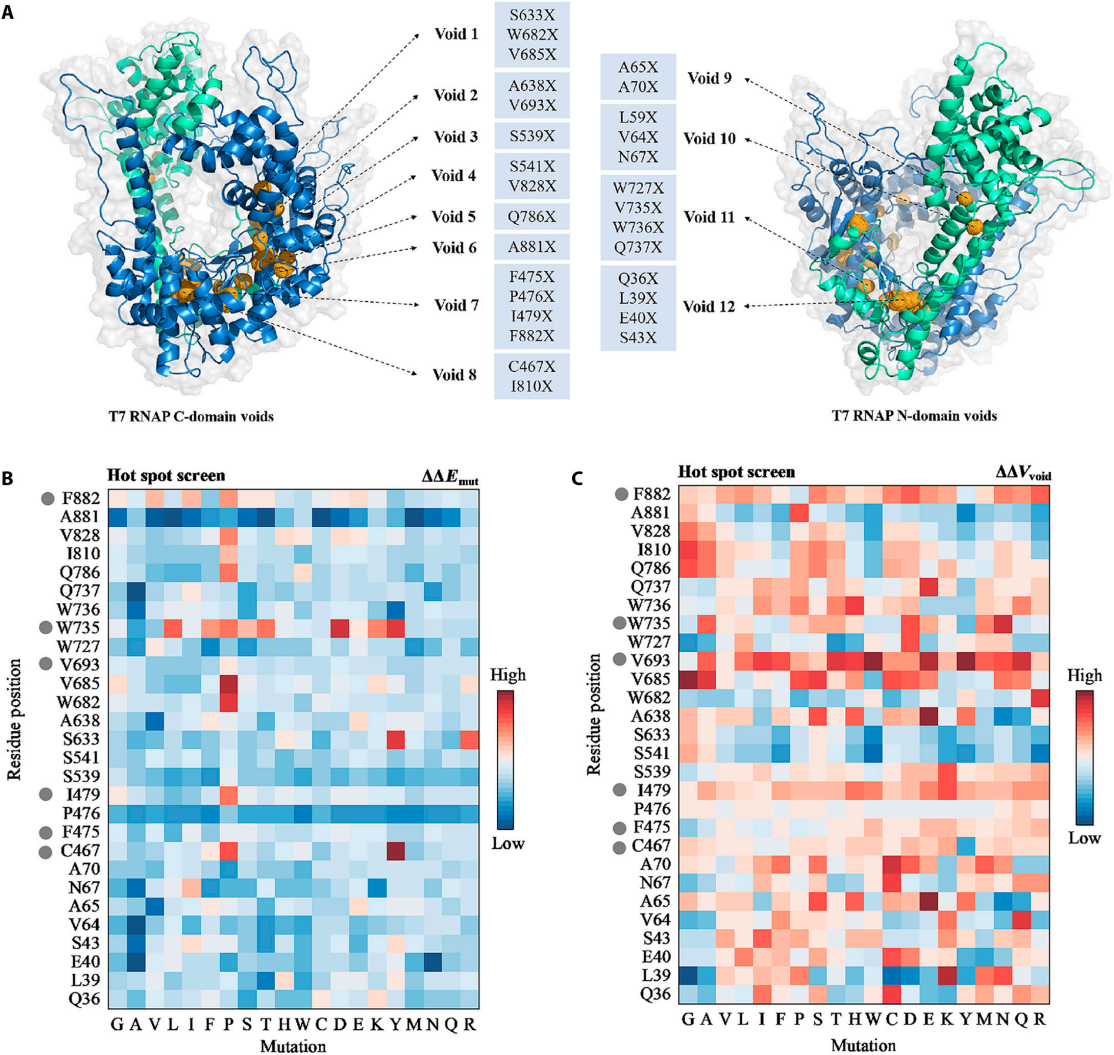
Semirational design of T7 RNAP mutagenesis libraries based on solvent-inaccessible region analysis. (A) Mutagenesis libraries were focused on solvent-inaccessible cavities to reduce dsRNA by-products and enhance catalytic activity. Structural analysis identified a total of 12 cavities and 29 hotspot residues. The N-terminal domain is colored green, and the C-terminal domain is colored blue. (B and C) Molecular dynamics simulations were used to analyze energetic and volume changes in single-site saturation mutants of T7 RNAP. Changes in unfolding free energy (ΔΔ*E*_mut_, in Rosetta energy units) were estimated using the Rosetta Cartesian_ddG protocol. Volume changes of the cavities (Δ*V*_cav_) were calculated using ProteinVolume v1.3, and ΔΔ*V*_cav_ is defined as the change in Δ*V*_cav_ (percentage of total molecular volume) upon mutation relative to the wild type. Sites that did not meet both criteria—ΔΔ*V*_cav_ range ≥ 0.06 and average ΔΔ*E*_mut_ < 8—were excluded from the final library (gray circles).

We modeled 4 crystal structures of T7 RNAP in both the initiation complex (IC; Protein Data Bank [PDB] IDs: 1CEZ, 2PI4, and 1QLN) and the elongation complex (EC; PDB IDs: 1MSW, 1H38, and 1S76), and docked a DNA template along with an RNA product to simulate their conformations during catalysis. These simulations identified 8 cavities in the C-terminal domain (cavity 1 to cavity 8) and 4 additional cavities in the N-terminal domain (cavity 9 to cavity 12) (Fig. 2A). We performed virtual mutations on key residues within each cavity using ProteinVolume v1.3 and estimated the change in unfolding free energy for each mutation with Rosetta Cartesian_ddG (Δ*E*_mut_) (Fig. 2B). Residues exhibiting low unfolding free energy and high cavity volume variability (∆∆*V*_cav_) were considered thermodynamically sensitive and prone to core packing disruption. We therefore focused on cavity residues meeting both criteria, particularly those with ∆∆*V*_cav_ range > 0.06 and average ∆∆*E*_mut_ < 8, to ensure candidate variants retained high activity at increased temperatures. Sixteen residue positions (P476, S539, S633, A638, W682, V685, Q786, Q36, L39, E40, S43, A65, A70, L59, V64, and N67) were selected to construct 5 combinatorial mutant libraries targeting potentially functional cavity-regulating sites (Fig. 2B).

**Fig. 3.**
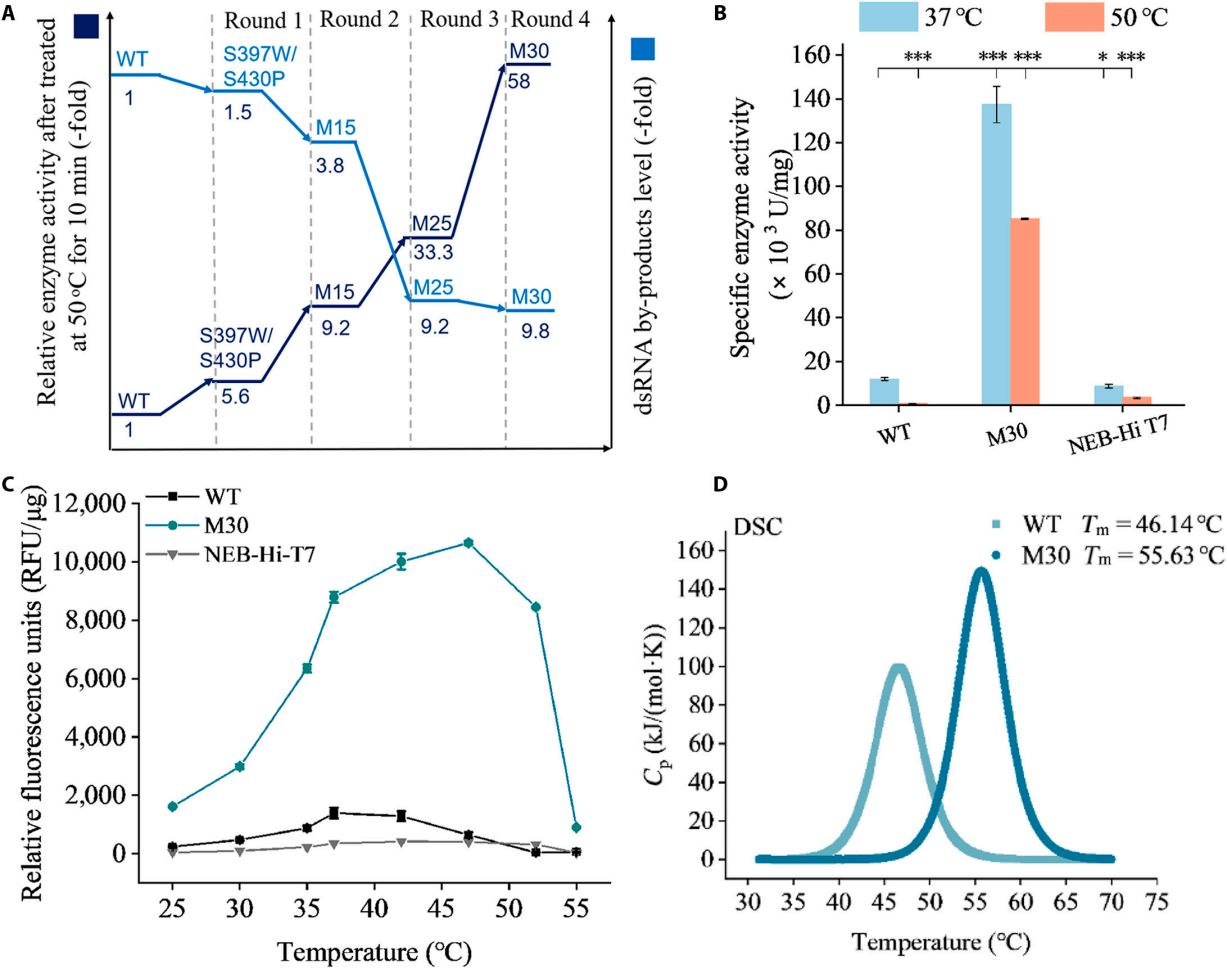
Enzymatic characterization of the M30 variants. (A) Evolutionary trajectory of T7 RNAP variants. The dark blue path represents the relative enzymatic activities of the variants compared with the wild type, while the light blue path represents the relative levels of dsRNA by-products. (B) Specific activities of different T7 RNAP variants at 37 and 50 °C. Error bars indicate the standard error of the mean from 3 independent experiments. Statistical analysis: *t* test; ***P < 0.001, *P < 0.05. (C) Relationship between temperature and enzyme activity of different T7 RNAP variants. Error bars indicate the standard deviation of 3 replicates. (D) Melting temperatures (*T*_m_) of T7 RNAP variants determined using differential scanning calorimetry.

In the second round of screening, 3 CAST mutant libraries were designed based on the spatial proximity of target residues in the C-terminal domain: P476X/S539X, I810X/A818X/A881X, and S633X/W682X/V685X/A638X/Q786X, using S397W/S430P as the template. The libraries were pooled and screened together (~3 × 10^8^ clones), leading to the identification of 4 variants (S397W/S430P/V685A, S397W/S430P/Q786L, S397W/S430P/Q786M, and S397W/S430P/S633P/Q786M) with improved relative activity following heat pretreatment (Table S1). Among these, M15 (S397W/S430P/S633P/Q786M) performed best, exhibiting a 9.42-fold increase in relative activity after heat pretreatment (Table S1) and a 3.8-fold reduction in dsRNA by-products in its transcribed RNA compared with the WT (Fig. 3A).

To further reduce dsRNA by-products and enhance T7 RNAP activity at increased temperatures, we constructed 2 additional libraries in the third screening round based on M15, targeting residues within the N-terminal cavities: A65X/A70X/L59X/V64X/N67X and Q36X/L39X/E40X/S43X. Screening approximately 7 × 10^7^ clones from the pooled libraries led to the identification of 4 improved mutants (M15/S43L, M15/S43R, M15/S43Y, and M15/S43E, Table S1). The best-performing variant, M25 (M15/S43E), exhibited a 9.2-fold reduction in dsRNA by-products and a 33.3-fold increase in relative activity after heat pretreatment compared with the WT (Table S1).

To further enhance M25, we individually introduced previously reported mutation sites (G47A, 884G, G47W, or Q744R), which are associated with reduced dsRNA formation or increased activity, into the M25 background for the fourth screening round [24]. Despite their previously reported benefits, incorporating G47A, 884G, or G47W into the M25 scaffold likely overconstrained the polymerase structure, perturbing the conformational plasticity required for template translocation and thereby caused a substantial loss of catalytic activity after heat pretreatment. Ultimately, we selected M30 (S43E/S397W/S430P/S633P/Q744R/Q786M), which exhibited a 9.8-fold reduction in dsRNA by-products and a 58-fold increase in activity after heat pretreatment compared with the WT (Fig. 3A and Table S2).

### Characterization of T7 RNAP variants

To better understand the catalytic performance of the M30 variant, we measured its specific activity and compared it with the WT enzyme and NEB-Hi-T7 RNAP, a commercial variant reported to reduce dsRNA at increased temperatures. At 37 °C, M30 exhibited approximately 10-fold and 15.6-fold higher specific activity than WT and NEB-Hi-T7, respectively. At 50 °C, its specific activity increased by ~113-fold and 25-fold, respectively (Fig. 3B), demonstrating a clear catalytic advantage over both WT and NEB-Hi-T7 across the temperature range of 37 to 52 °C (Fig. 3C).

M30 also displays enhanced thermostability, with a half-life at 50 °C approximately 283-fold longer than that of WT. Its T_50_^15^ value is 9.5 °C higher, and its melting temperature (*T*_m_) is 9 °C higher than WT (Table S2 and Fig. 3D). The enthalpy change (Δ*H*_m_) for M30 is ~903 kJ/mol, compared with 636 kJ/mol for WT, indicating a more stable network of intramolecular interactions in the folded state (Fig. S8).

**Fig. 4.**
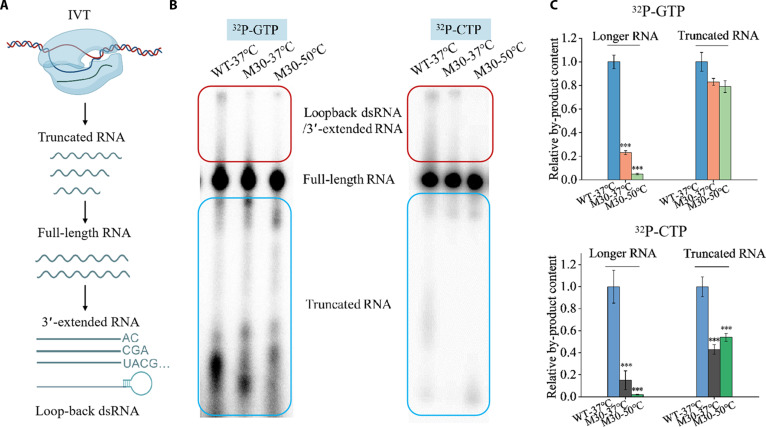
Denaturing PAGE analysis of radiolabeled RNA products transcribed by WT-T7 RNAP and M30. (A) Schematic diagram of RNA products generated by wild-type T7 RNAP during in vitro transcription. (B) Radiolabeled RNA products generated at 37 and 50 °C by WT T7 RNAP or M30 were analyzed by denaturing PAGE. Reactions containing α-^32^P-GTP or α-^32^P-CTP selectively labeled species, including truncated products (blue panels), full-length RNA, 3′-extended RNA, and loopback transcription products (red panels). (C) Relative levels of by-products from the radioactive sequencing gels using α-^32^P-GTP or α-^32^P-CTP. Error bars represent the standard error of the mean from 3 independent experiments. Statistical analysis: *t* test; significance compared with WT at 37 °C: ***P < 0.001.

It is well established that dsRNA arises from aberrant elongation and is closely related to the 3′-end homogeneity of RNA transcripts (Fig. 4A) [21]. To assess M30’s performance in minimizing dsRNA formation, we measured its 3′-end homogeneity using an RNase T1 digestion assay and compared it with WT and NEB-Hi-T7 (Fig. S9A) [25]. At 37 °C, M30 showed markedly improved 3′-end homogeneity (60%) compared with WT (5.6%). Enhanced 3′-end homogeneity is critical for producing transcripts with consistent translation efficiency and reduced innate immune activation, underscoring the pharmaceutical relevance of M30’s improved transcript quality. Furthermore, M30 maintained higher 3′-end homogeneity than NEB-Hi-T7 at both 37 and 50 °C, whereas NEB-Hi-T7 exhibited <5% 3′-end homogeneity under both conditions (Fig. S9B).

To further evaluate the enhanced 3′-end homogeneity of M30, isotopic labeling was used to analyze its RNA products. At 37 °C, the levels of 3′-extended products and dsRNA by-products were reduced by ~4-fold compared with WT, and decreased even further at 50 °C (Fig. 4B and C). These results are consistent with the improvements observed in the RNase T1 digestion assay and suggest that increased temperatures further suppress dsRNA by-product formation.

### Biophysical assays and molecular mechanisms of improved variants

**Fig. 5.**
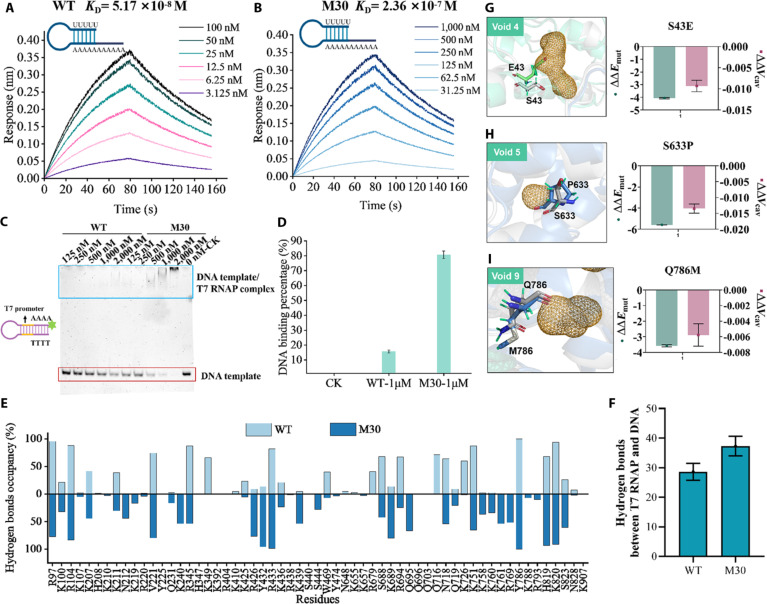
Biophysical assays and molecular mechanisms of the M30 variant. (A and B) Multi-concentration association–dissociation curves of RNA substrate binding to T7 RNAP. The blue hairpin represents the RNA substrate. (C) Binding affinity of WT and M30 variants to the DNA template measured by EMSA. (D) DNA-bound fraction of WT and M30 at 1 μM enzyme concentration from panel (C). The percentage of bound DNA was calculated as the reduction in unbound DNA relative to the no-protein control (CK). (E) Occupancy of hydrogen bonds formed between T7 RNAP residues and the DNA template. The histogram shows the percentage of simulation time during which individual residues form hydrogen bonds with DNA, based on the equilibrated portion of the molecular dynamics trajectory. These residues are critical for specific recognition and stable DNA binding. (F) Number of hydrogen bonds formed between T7 RNAP and the DNA template during the stable equilibrium phase of the molecular dynamics simulation. (G to I) Structural details of amino acids and voids at positions 43, 633, and 786 (cavity 4, cavity 5, and cavity 9) in WT and M30 variants. Energy differences (ΔΔ*E*_mut_, in Rosetta energy units) and changes in cavity volume (∆∆*V*_cav_) are shown. Error bars represent the standard error of the mean from 3 independent experiments.

Given the observed reduction in dsRNA by-products and improved 3′-end homogeneity of M30, we hypothesized that the variant may alter its interactions with nucleic acid substrates to favor canonical transcription. We measured the binding affinities of M30 and WT to RNA and DNA templates. A chimeric RNA forming a partial hairpin was designed to mimic a dsRNA precursor structure (Fig. 5A). Bio-layer interferometry revealed that M30 exhibited a 4.56-fold higher dissociation constant (*K*_D_) for RNA compared with WT (2.36 × 10^−7^ M vs. 5.17 × 10^−8^ M) (Fig. 5A and B), indicating weakened RNA-binding affinity. In contrast, electrophoretic mobility shift assays (EMSAs) using a 5′-6-FAM-labeled 43-bp DNA hairpin containing a T7 promoter showed that M30 had enhanced DNA binding affinity, with a 4.5-fold higher binding signal than WT at 1 μM. These results indicate that M30 disfavors RNA re-binding (with ~20-fold lower affinity for RNA versus DNA) while reinforcing DNA engagement, providing a kinetic basis for reduced dsRNA formation and improved 3′-end homogeneity.

To elucidate the structural basis for this altered substrate preference, we performed MD simulations. Compared with WT, M30 formed 10 additional hydrogen bonds with the DNA template (Fig. 5E and F), particularly involving residues W397 and P430, which established persistent polar interactions with the surrounding DNA backbone (Fig. S10). These simulations suggest that M30 stabilizes the transcription complex through stronger DNA interactions. The free energy barrier (∆*G*) affects both enzyme catalytic efficiency and conformational dynamics [26]. Three states (IC, intermediate complex [IM], and EC) were analyzed for ∆*G* in WT and M30. M30 exhibited a ∆*G* reduction of 82.228 Rosetta energy units (REU) in IM (WT: 31,824.316 REU vs. M30: 31,740.088 REU) and 6.992 REU lower ∆*G* in EC (WT: 6,703.454 REU vs. M30: 6,696.462 REU) (Fig. S11), indicating a faster transition from IC to EC. The free energy landscape (FEL) maps protein conformational dynamics and thermodynamic stability through variations in free energy during simulations [27]. The FEL analysis predicted that M30 exhibits greater conformational rigidity in the lowest-energy states compared with WT, with ~0 to 12 for M30 versus ~0 to 16 for WT in the IM, and ~0 to 10 for M30 versus ~0 to 12 for WT in the EC (Fig. S12). These simulations suggest that the M30 variant may enhance transcriptional elongation efficiency by lowering energetic barriers and stabilizing the transcription complex.

A detailed analysis of the local conformation predicted that S43E (∆∆*V*_cav_ = –0.0093, ∆∆*E*_mut_ = –4.06), S633P (∆∆*V*_cav_ = –0.01348, ∆∆*E*_mut_ = –5.58), and Q786M (∆∆*V*_cav_ = –0.0132, ∆∆*E*_mut_ = –4.06) decreased the volume of cavity 4, cavity 5, and cavity 9, respectively (Fig. 5G to I). These findings are consistent with previous studies showing that reduced cavity volume correlates with lower mutational free energy.

### Application of M30 in mRNA production

**Fig. 6.**
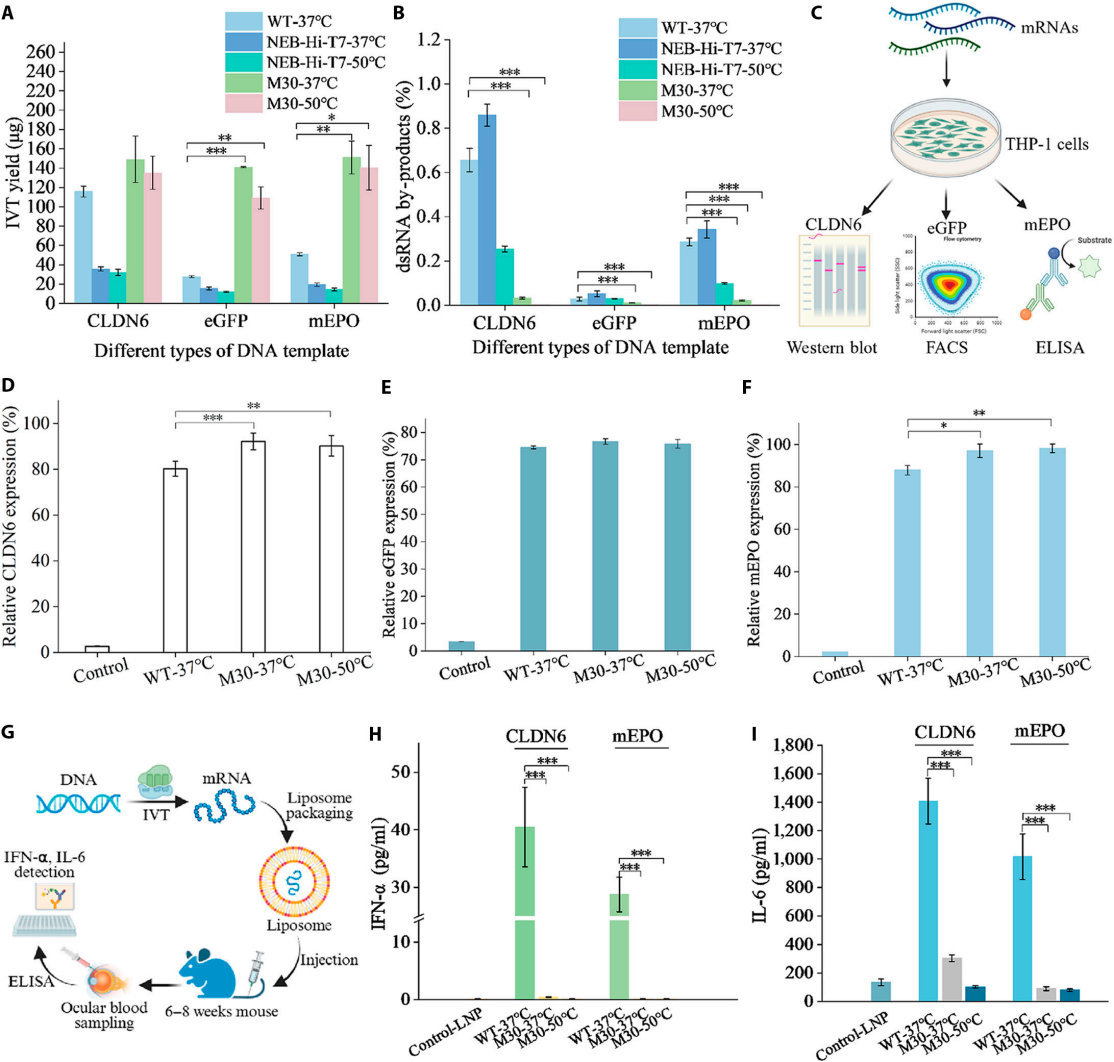
Comparative analysis of the biological effects of mRNA products transcribed by WT and M30 variants. (A) IVT yields of mRNAs transcribed by different T7 RNAP variants using various DNA templates at 37 or 50 °C. (B) Quantification of dsRNA concentrations in crude IVT reactions using ELISA. (C) Expression levels of different mRNAs transcribed by T7 RNAP variants were measured using appropriate detection methods. (D) Comparison of CLDN6 protein expression from mRNAs transcribed by M30 or WT in THP-1 cells. (E) Comparison of EGFP protein expression from mRNAs transcribed by M30 or WT in THP-1 cells. (F) Comparison of MEPO protein expression from mRNAs transcribed by M30 or WT in THP-1 cells. (G) Workflow for measuring inflammatory cytokines in mouse serum. (H) Three mRNAs synthesized by WT or M30 were encapsulated to form mRNA LNP, which was further injected into the tail vein of mice. Serum was collected after 6 h, and IFN-α levels were detected by ELISA. (I) Three different mRNAs synthesized by WT or M30 were encapsulated to form mRNA LNP, which was further injected into the tail vein of mice. Serum was collected after 6 h, and IL-6 levels were detected by ELISA. Error bars represent the standard error of the mean from 4 independent experiments. Statistical analysis: *t* test; ***P < 0.001, **P < 0.01, *P < 0.05.

To evaluate the advantages of the M30 variant in mRNA production, we compared the performance of its RNA products with those of WT and the commercial variant NEB-Hi-T7. We utilized 3 DNA templates: *egfp* [28], *mepo* [29], and *cldn6* [30]. In a short time, at IVT reactions of 20 min, M30 achieved increased yields ranging from 1.3- to 5.6-fold compared with WT at both 37 and 50 °C (Fig. 6A). In contrast, NEB-Hi-T7 consistently produced lower yields than WT under the same conditions (Fig. 6A). To support M30’s feasibility for industrial-level applications, we carried out scale-up IVT experiments with reaction volumes up to 17.5 ml. In these reactions, the total mRNA yield exceeded 100 mg while maintaining low dsRNA levels (Table S4). We found that both mRNA yield and dsRNA content remained highly consistent across reaction volumes ranging from 20 μl to 17.5 ml, which indicates that our IVT system can be linearly scaled.

To assess the immune-stimulatory potential of T7 transcripts, we measured dsRNA by-products in crude IVT products from the different variants using a standard enzyme-linked immunosorbent assay (ELISA)-based assay. As illustrated in Fig. 6B, all 3 RNA products (mEPO, CLDN6, and GFP) transcribed by M30 exhibited substantially reduced dsRNA levels compared with WT. At 37 °C, dsRNA levels produced by M30 were less than 10% of those from WT across all DNA templates. Notably, at 50 °C, dsRNA by-products from M30 dropped to just 0.1%, reaching as low as 0.001 ng/μg. In contrast, NEB-Hi-T7 showed a 2- to 4-fold reduction in dsRNA levels at 50 °C relative to 37 °C, consistent with previous reports [17].

To evaluate whether mRNAs transcribed by M30 function efficiently in cells, we measured protein expression levels of CLDN6, mEPO, and eGFP mRNAs generated by M30 and WT T7 RNAP (Fig. 6C). The mRNA templates were selected to represent distinct therapeutic categories with differences in transcript length, sequence complexity, and application scenarios, including reporter, immunotherapy-related, and protein replacement mRNAs. CLDN6 mRNA transcribed by M30 produced 16% and 14% higher protein expression at 37 and 50 °C, respectively, compared with WT (Fig. 6D and Fig. S13), indicating that reducing dsRNA by-products enhances CLDN6 protein expression (Fig. 6B). However, further lowering dsRNA levels from 0.03% to 0.003% did not significantly affect CLDN6 expression, supporting the “functional threshold” principle in dsRNA regulation, where complete elimination is unnecessary and trace amounts may be required for optimal translation. For eGFP, no substantial differences in RNA quantity or protein expression were observed among mRNAs generated by the different T7 RNAPs, likely due to the relatively low dsRNA levels produced by WT (Fig. 6B and E). For mEPO, protein expression from M30-transcribed mRNA was slightly higher than WT at both 37 and 50 °C (Fig. 6F).

To simulate vaccine administration, we delivered various mRNAs, crudely purified by LiCl precipitation, into mice using liposomal packaging. Serum concentrations of interleukin-6 (IL-6) and interferon-α (IFN-α) were subsequently measured (Fig. 6G) [31,32]. IFN-α was chosen as a sensitive marker of dsRNA-triggered innate immune sensing, while IL-6 reflects broader acute inflammatory responses, together providing complementary readouts of mRNA immunogenicity and tolerability in vivo. As expected, mRNAs produced by WT T7 RNAP triggered strong IL-6 and IFN-α responses (Fig. 6H and I). In contrast, mRNAs transcribed by M30 at both 37 and 50 °C induced minimal IL-6 and IFN-α responses, consistent with the low levels of dsRNA produced by the M30 variant.

RNA polymerase fidelity is critical to prevent errors during transcription. We therefore measured the error rates of M30 and WT using next-generation sequencing. The results showed no significant difference (WT: 1.28 × 10^−5^ errors/base; M30: 1.25 × 10^−5^ errors/base) (Fig. S14). Consistently, intracellular protein expression data confirmed that mRNAs transcribed by M30 function efficiently within cells.

## Discussion

In this study, we developed a T7 RNAP variant, M30, with markedly improved enzymatic properties. M30 exhibits approximately a 10-fold increase in catalytic efficiency at 37 °C, a substantial reduction in dsRNA by-products, and enhanced thermostability. Compared with the commercially available NEB-Hi-T7, M30 demonstrates both higher catalytic activity and dramatically lower dsRNA levels under equivalent reaction conditions. Moreover, relative to the previously reported G47A+884G variant, M30 shows superior catalytic efficiency. Functionally, mRNAs synthesized by M30 support robust protein expression in human cells and in vivo while eliciting minimal innate immune responses.

The therapeutic potential of mRNA continues to expand, particularly in cancer therapeutic vaccines, protein replacement therapies, and in vivo CAR-T applications, each of which poses distinct challenges for RNA transcript quality and immunogenicity. Tumor vaccines require rapid, high-yield synthesis of individualized antigens with minimal innate immune activation to avoid suppressing adaptive responses [33]. Protein replacement therapies often involve repeated dosing, requiring highly pure mRNA with low dsRNA by-products to minimize immunotoxicity [34,35]. In vivo CAR-T applications demand efficient production of long, structured transcripts that preserve 3′-end integrity for consistent expression [36]. M30’s enhanced transcriptional efficiency, reduced dsRNA by-products, and improved 3′-end homogeneity directly address these challenges, highlighting its potential as a next-generation RNA synthesis tool for both clinical and manufacturing applications.

Biophysical assays and molecular mechanism analyses revealed that M30 exhibits enhanced DNA-template affinity and reduced RNA-template affinity. This shift in binding selectivity is particularly important because, although T7 RNAP is a DNA-dependent RNA polymerase, it can engage RNA as an alternative template when full-length transcripts accumulate, leading to self-priming or reannealing with abortive transcripts and resulting in 3′-end heterogeneity and dsRNA by-products [21,37,38]. The M30 variant appears to reprogram the enzyme template selection landscape, promoting canonical promoter-initiated transcription while suppressing aberrant extension events. Notably, the responsible mutations are located in buried, non-catalytic regions, indicating that subtle conformational adjustments can modulate template usage without perturbing the catalytic core. This mechanism introduces “template selectivity” as a novel engineering principle for improving transcriptional fidelity and efficiency in T7 RNAP. Previous engineering efforts on T7 RNAP have primarily relied on empirical strategies, such as introducing additional amino acid residues in the C-terminal “foot” region or performing saturation mutagenesis at selected functional sites, to reduce dsRNA by-product formation [39,40]. While these approaches have been shown to be effective at the phenotypic level, the underlying mechanistic basis for dsRNA suppression has not been explicitly addressed. In contrast, our experimental results and mechanistic analyses indicate that a key, previously underappreciated factor contributing to reduced dsRNA formation is the modulation of the enzyme’s relative affinity toward DNA versus RNA templates. Rather than simply altering local structural elements, the engineered variant exhibits a shifted template preference that disfavors RNA-mediated parasitic transcription while maintaining productive DNA-templated transcription. This finding highlights “template selectivity” as a mechanistic principle that complements existing enzyme engineering strategies and provides a more rational framework for suppressing undesired side-reaction pathways.

In conclusion, our Ab-FADS-based ultrahigh-throughput screening platform allowed the evaluation of up to 10^8^ variants per day, accelerating the directed evolution of T7 RNAP. The resulting M30 variant enables rapid, high-yield mRNA synthesis with reduced dsRNA by-products and minimal immunogenicity. Moreover, M30 reveals “template selectivity”, driven by altered DNA- and RNA-binding preferences, as a novel parameter for T7 RNAP engineering. This insight not only advances therapeutic mRNA production but also expands the conceptual framework for future polymerase optimization. Future work should evaluate M30’s performance across large-scale process robustness and modified nucleotide chemistries to ensure broad applicability in industrial mRNA platforms.

## Materials and Methods

### Oligonucleotides

All primers and DNA oligonucleotides used for mutant library construction, IVT, and binding affinity assays were synthesized by Genewiz (Azenta), with sequences provided in Table S3 and the Supporting Sequence in the Supplementary Materials.

### Construction of a random mutagenesis library

The random mutagenesis library was generated using error-prone PCR, with mutation rates controlled by varying the concentration of manganese ions. The reaction contained DreamTaq (0.05 U μl^−1^), DreamTaq buffer (Thermo Fisher Scientific), dATP (250 μM), dGTP (250 μM), dCTP (1,050 μM), dTTP (1,050 μM), T7 RNAP-F (forward primer, 0.4 μM), T7 RNAP-R (reverse primer, 0.4 μM), T7 RNAP gene template (0.2 ng μl^−1^), and MnCl_2_ (0.2 to 0.8 mM). The mixture was divided into 25-μl reactions and subjected to PCR under the following conditions: 95 °C for 3 min (1 cycle); 95 °C for 15 s, 55 °C for 30 s, 72 °C for 90 s (30 cycles); and a final extension at 72 °C for 5 min (1 cycle). The purified PCR products were digested with *Sac*I and *Hin*dIII and ligated into the pQE-80L vector using T4 ligase. Recombinant vectors were transformed into *E. coli* 5G cells via electroporation, and clones were screened to determine mutation frequency. The mutant library generated at 0.4 mM MnCl_2_, which exhibited an average of 2 to 3 mutations per gene, was selected.

### Structural analysis and design of semirational design libraries

Based on the crystal structures of the IC (PDB IDs: 1CEZ, 1QLN, and 2PI4) and the EC (PDB IDs: 1H38, 1MSW, and 1S76) of T7 RNAP, missing loops and side chains were reconstructed using the RosettaCM comparative modeling protocol [41]. DNA and RNA were reintroduced into the rebuilt protein structures by aligning the original complex proteins with the reconstructed apo-proteins and transferring nucleic acid coordinates to the reconstructed structures. The resulting DNA/RNA–T7 RNAP complexes were subsequently minimized under heavy-atom crystallographic constraints using the Rosetta constraint minimization protocol. Fixed-backbone folding free energy changes (∆∆*E*_mut_) induced by mutations were calculated in REU using the Rosetta Cartesian_ddG method [42,43]. Changes in protein void volumes were determined using ProteinVolume software [44]. For all mutant structures, solvent-excluded volume, van der Waals volume, and void volume were computed with ProteinVolume v.1.3 using default parameters. Further details are provided in the Supplementary Methods. All computational analyses were performed on the Shanghai Jiao Tong University Center for High-Performance Computing platform.

### Construction of T7 RNAP combinatorial mutant libraries

The design principle of the combinatorial mutant libraries is illustrated in Fig. S2. Each 200-bp oligonucleotide was designed to contain 1 or 2 mutation sites. Gene fragments with 25-bp overlapping sequences at both ends were initially amplified by PCR. The 50-μl PCR reaction contained 1× PrimerSTAR Max, 0.2 mM dNTP mix, 50 ng T7 RNAP mutant plasmid, 0.4 μM oligo fragment, and 0.4 μM complementary overlapping primers. PCR products were treated with *Dpn*I to digest the plasmid template and then purified to obtain diverse gene fragment products. Overlap extension PCR was subsequently performed to reassemble full-length genes. The 50-μl reaction contained 25 μl of 2× PrimerSTAR Max, 100 ng of equimolar mixed gene fragments, 50 ng of T7 RNAP mutant plasmid, 0.4 μM T7 RNAP-F, and 0.4 μM T7 RNAP-R. The PCR products were treated with *Dpn*I to remove template plasmid, purified, digested with *Sac*I and *Hin*dIII, and ligated into the pQE-80L vector using T4 ligase. Recombinant vectors were transformed into *E. coli* 5G cells via electroporation, and clones were screened to evaluate library quality.

### Screening schematic of mutant libraries using the FADS system

The T7 RNAP libraries were electroporated into 100 μl of BL21(DE3) competent cells, followed by the addition of 400 μl of LB medium and incubation at 37 °C, 220 rpm for 1 h. The culture was then transferred to 9 ml of LB containing 50 μg/ml ampicillin and incubated at 37 °C, 220 rpm until OD_600_ reached ~0.6. Expression was induced by adding 1 mM IPTG for 6 h at 37 °C. The induced culture was washed twice with 1× PBS (pH 7.5) and resuspended in 200 mM HEPES (pH 7.5) to adjust OD_600_ to 0.05. The cell suspension and substrate-lysis solution—containing 400 mM HEPES (pH 7.5), 60 mM MgCl_2_, 8 mM NTP mix, 40 mM dithiothreitol, 0.4 U/μl RNase inhibitor, 200 μM DFHBI, 1 μM 4iSp DNA template, and BugBuster (Novagen, 50% v/v)—were co-encapsulated at a 1:1 ratio in 30 μm of water-in-oil droplets using a droplet-making device. The droplets were incubated at 45 °C for 15 min, followed by 37 °C for 2 h. The enzymatic reaction was terminated on ice, and the droplets were re-injected into the detection/sorting device (CytoSpark MSP Droplet Sorting System, Dapu Biotechnology Co., Ltd.). Droplets exceeding the screening threshold were collected into 1.5-ml microcentrifuge tubes. Positive droplets were emulsified, and the corresponding genes were recovered by PCR. The PCR products were digested with *Sac* I and *Hin*d III, cloned into the pQE-80L vector, and transformed into BL21(DE3) cells for subsequent screening.

Secondary screening was performed using the T-STAR system in a 96-well plate. Briefly, the T-STAR reaction mixture contained 500 nM DNA template encoding the iSpinach aptamer, 200 mM HEPES (pH 7.5), 40 mM MgCl_2_, 5 mM NTP mix (or modified NTPs), 20 mM dithiothreitol, 0.2 U/μl RNase inhibitor, and 100 μM DFHBI. Ten microliters of crude enzyme lysate from different T7 RNAP mutants were incubated at 45 °C for 15 min and then immediately placed on ice. One microliter of the treated crude enzyme was added to the T-STAR system, and reactions were incubated at 37 °C for 20 min, with fluorescence measured at an excitation of 470 nm and emission of 512 nm using a microplate reader (SpectraMax M4, Sunnyvale). Mutants exhibiting fluorescence intensities 1.5-fold higher than WT were selected, and mutation sites were confirmed by DNA sequencing.

### Biolayer interferometry

The binding affinity of the single-stranded chimeric RNA chain (AAAAAAAAAAAAAAAAAAAA-dCdCdCdCdC-UUUUUUUUUU) to WT and M30 variant was measured by biolayer interferometry using a ForteBio Octet K2 system. All assays were performed at 30 °C with continuous shaking at 1,000 rpm. The assay buffer consisted of PBS supplemented with 0.1% bovine serum albumin, 0.01% Tween-20, and 1% dimethyl sulfoxide. Biotinylated T7 RNAP protein (50 μg/ml) was immobilized on Super Streptavidin (SSA) biosensors. RNA was dissolved in DEPC-treated water, heated at 75 °C for 5 min, and then slowly cooled to 25 °C to fold into a hairpin structure. Sensors were washed with assay buffer for 10 min after each association and dissociation cycle to remove nonspecifically bound protein and establish a baseline. Raw kinetic data were acquired using Data Acquisition software (ForteBio), and association/dissociation rate constants (*k*_on_/*k*_off_) were calculated using the double reference subtraction method in Data Analysis software (ForteBio). Binding affinities (*K*_D_) were determined following the standard protocol.

### Electrophoretic mobility shift assay

A 5′-6-FAM-labeled 43-bp DNA hairpin was heated to 85 °C for 5 min and then cooled to 25 °C over 20 min to allow secondary structure formation. Binding reactions (40 mM Tris-HCl, pH 7.5, 10 mM DTT, 30 mM MgCl_2_, and 10 nM DNA hairpin) were prepared with serial dilutions of WT or M30 enzymes (0 nM, 125 nM, 250 nM, 500 nM, and 1 μM) and incubated at 37 °C for 20 min. Reactions were terminated by adding 1× EMSA/Gel-shift loading buffer, and protein–DNA complexes were resolved on 6% native PAGE gels. Gels were imaged using a ChemiScope 6000 Imaging System (Clinx), and images were analyzed with ImageJ. The percentage of bound DNA was calculated as the reduction of unbound DNA relative to the no-protein control (CK).

### mRNA preparation and dsRNA by-product quantification

Linear DNA templates encoding eGFP, CLDN6, or mEPO with poly(A) tails were generated by *Bsp*Q I digestion of plasmids containing the respective genes. For IVT yield quantification, a 20-μl IVT reaction containing 40 mM HEPES (pH 7.8); 40 mM MgSO₄; 1.5 mM TCEP; 10 mM each of ATP, GTP, CTP, and N1-Me-pUTP; 100 nM T7 RNA mutant; and 1 μg DNA template was incubated at 37 or 50 °C for 20 min. RNA products were treated with DNase I and purified via LiCl precipitation. The resulting pellet was washed twice with pre-cooled 70% ethanol and dissolved in RNase-free H_2_O. RNA concentration was determined using a NanoDrop 2000 (Thermo Fisher Scientific).

For dsRNA quantification, co-transcriptional capping was performed in a reaction containing 40 mM HEPES (pH 7.8); 40 mM MgSO₄; 1.5 mM TCEP; 10 mM each of ATP, GTP, CTP, and N1-Me-pUTP; 100 nM T7 RNA mutant; 8 mM cap1 (3′OH AG); 1 μg of DNA template; 0.002 U inorganic pyrophosphatase; 0.5 U/μl Vaccinia Capping Enzyme; 2.5 U/μl mRNA Cap 2′-O-Methyltransferase; and 1 U/μl recombinant RNase inhibitor, incubated at 37 °C for 2 h. DNA digestion and RNA purification were performed as described above. dsRNA quantification was conducted using a commercial Quantitative dsRNA Detection Kit (ELISA) (Hzymes). All the dsRNA quantification was performed using the ELISA kit. Four replicates per sample were diluted to fall within the standard curve range. For each replicate, 100 μl of the dilution was added to the reaction wells and incubated at 25 °C for 1 h. The supernatant was discarded, 100 μl of Detection Antibody was added, and incubation continued at 25 °C for 1 h. Absorbance at 450/650 nm was measured, and concentrations were calculated using a 5-parameter logistic regression curve in ELISACalc software.

### Intracellular protein expression analysis of transcribed mRNA

The human monocytic cell line THP-1 was seeded in 12-well plates (2.5 × 10^5^ cells/well) and cultured overnight. The capped *egfp*, *mepo*, and *cldn6* mRNAs were transfected into THP-1 cells using Lipofectamine MessengerMAX (Invitrogen) according to the manufacturer’s instructions. For eGFP detection, single-cell suspensions were analyzed by flow cytometry (BD Fortessa), and data were processed using FlowJo. For CLDN6 quantification, cell lysates were analyzed by Western blot using Claudin 6 Rabbit mAb (ABclonal) and anti-β-actin antibodies, with band intensities normalized to β-actin using ImageJ. For mEPO analysis, protein levels in the cell supernatant were measured using a Mouse EPO/Erythropoietin ELISA Kit (Proteintech) following the manufacturer’s instructions.

### Immune response assay of transcribed mRNA

Capped mRNA was encapsulated in LNPs via ethanol-drop nanoprecipitation using a microfluidic system (MicroFlow S, Mingtai Pharmaceutical Equipment) with an FLowTech S chip. Lipid components included SM-102, DSPC (AVT), cholesterol (AVT), and DMG-PEG2000 at a molar ratio of 50:10:38.5:1.5. Lipids and mRNA were mixed at a 1:3 molar ratio in 50 mM acetate buffer (pH 5.0) at a flow rate of 12 ml/min. Control LNPs were prepared without mRNA. Following formulation, LNPs were neutralized with Tris-HCl (pH 7.5), supplemented with sucrose, sterile-filtered, and characterized, exhibiting particle sizes of 50 to 100 nm, PDI < 0.2, and >90% encapsulation efficiency.

Mice were administered intravenously via the tail vein with lipid nanoparticle formulations containing *mEPO* or *CLDN6* mRNA at a dose of 0.5 mg/kg and euthanized 6 h post-injection. Eyes were enucleated, and blood was collected and centrifuged to obtain serum. IL-6 and IFN-α levels were measured using a Mouse IL-6 ELISA Kit (Proteintech) and a Mouse IFN-α ELISA Kit (Invitrogen), respectively, following the manufacturer’s instructions. All animal procedures were approved by the Institutional Animal Care and Use Committee of Shanghai Jiao Tong University and conducted in accordance with ethical guidelines.

## Data Availability

All data needed to evaluate the conclusions in the paper are present in the paper and/or the Supplementary Materials.
