# Supplementary material for: Directed Evolution of T7 RNA Polymerase Minimizes dsRNA By-product and Enables High-Fidelity mRNA Synthesis for Demanding Therapeutic Applications
Source: Research (Wash D C). 2026 Feb 25;9:1172. doi: 10.34133/research.1172 (PMC12932865; doi:10.34133/research.1172)
Supplement: Supplementary 1 — Figs. S1 to S14 Tables S1 to S4 [file research.1172.f1.docx]

**Supplementary Materials for**

**Directed evolution of T7 RNA polymerase minimizes dsRNA byproduct and enables high-fidelity mRNA synthesis for demanding therapeutic applications**

Weitong Qin, Ting Nie *et al.*

*Guangyu Yang. Email: yanggy@sjtu.edu.cn

1. **Supporting figures legend**


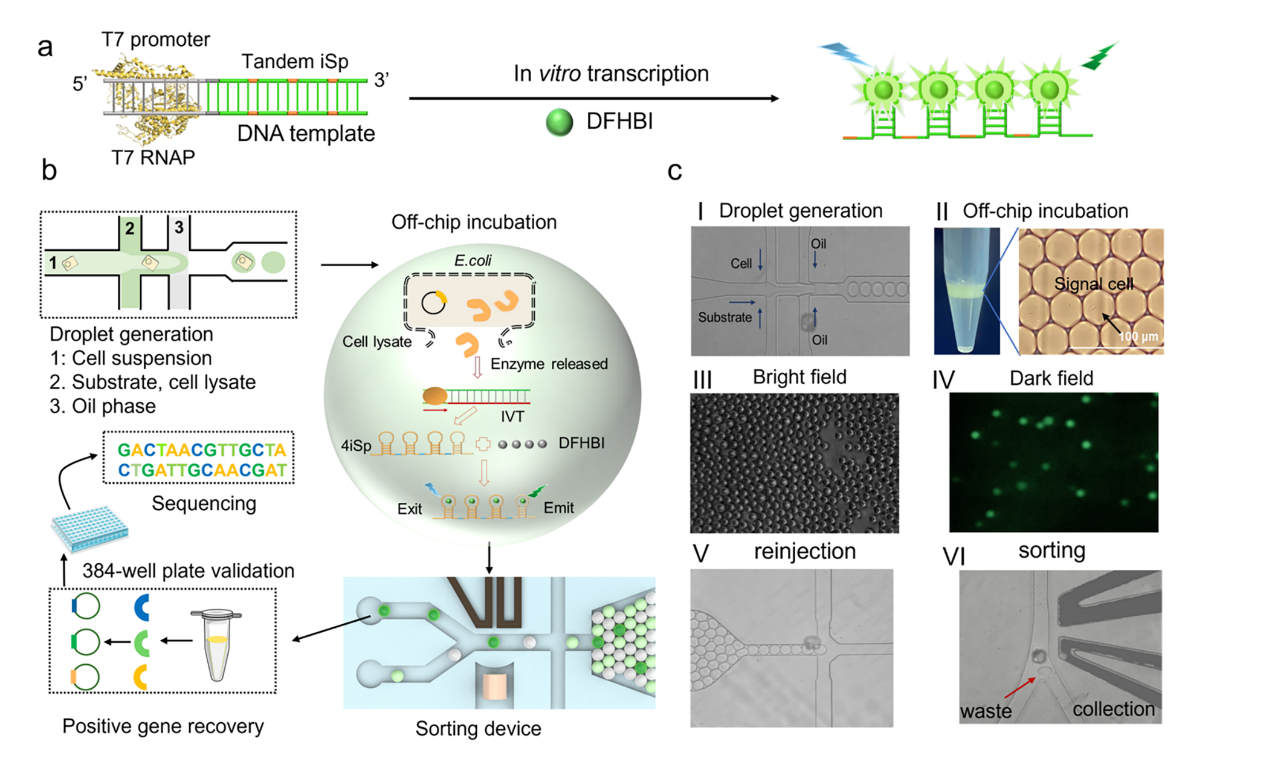


**Fig. S1. Schematic of aptamer-based FADS for T7 RNAP evolution. (a)** Principle of the STAR assay. The DNA template contains a T7 promoter and a tandem iSpinach aptamer. Transcription reactions are performed in a 384-well plate in the presence of T7 RNAP variants and DFHBI dyes (green balls). During in vitro transcription, only full-length transcripts bind DFHBI, generating a fluorescence signal. Fluorescence is continuously measured using a microplate reader, with relative fluorescence units (RFUs) proportional to the concentration of the target RNA and the activity of the T7 RNAP variants. **(b)** Schematic of the aptamer-based FADS (Ab-FADS) system. Cell suspensions expressing T7 RNAP variants and substrates (STAR system) are co-encapsulated in water-in-oil droplets. After 3 hours of off-chip incubation, droplets are re-injected into the sorting device. Positive genes are amplified by PCR, reconstructed into the vector, and transformed into *E. coli* BL21 (DE3). The activities of T7 RNAP variants are further validated using the STAR system, and variants with improved activity are sequenced. **(c)** Droplet generation (I), off-chip incubation (II), and observation of cell encapsulation rates under a microscope (III, IV). Arrows indicate single cells. Droplets are re-injected into the sorting device (V), and positive droplets (red arrow) are captured during sorting. The black region represents the electrode.


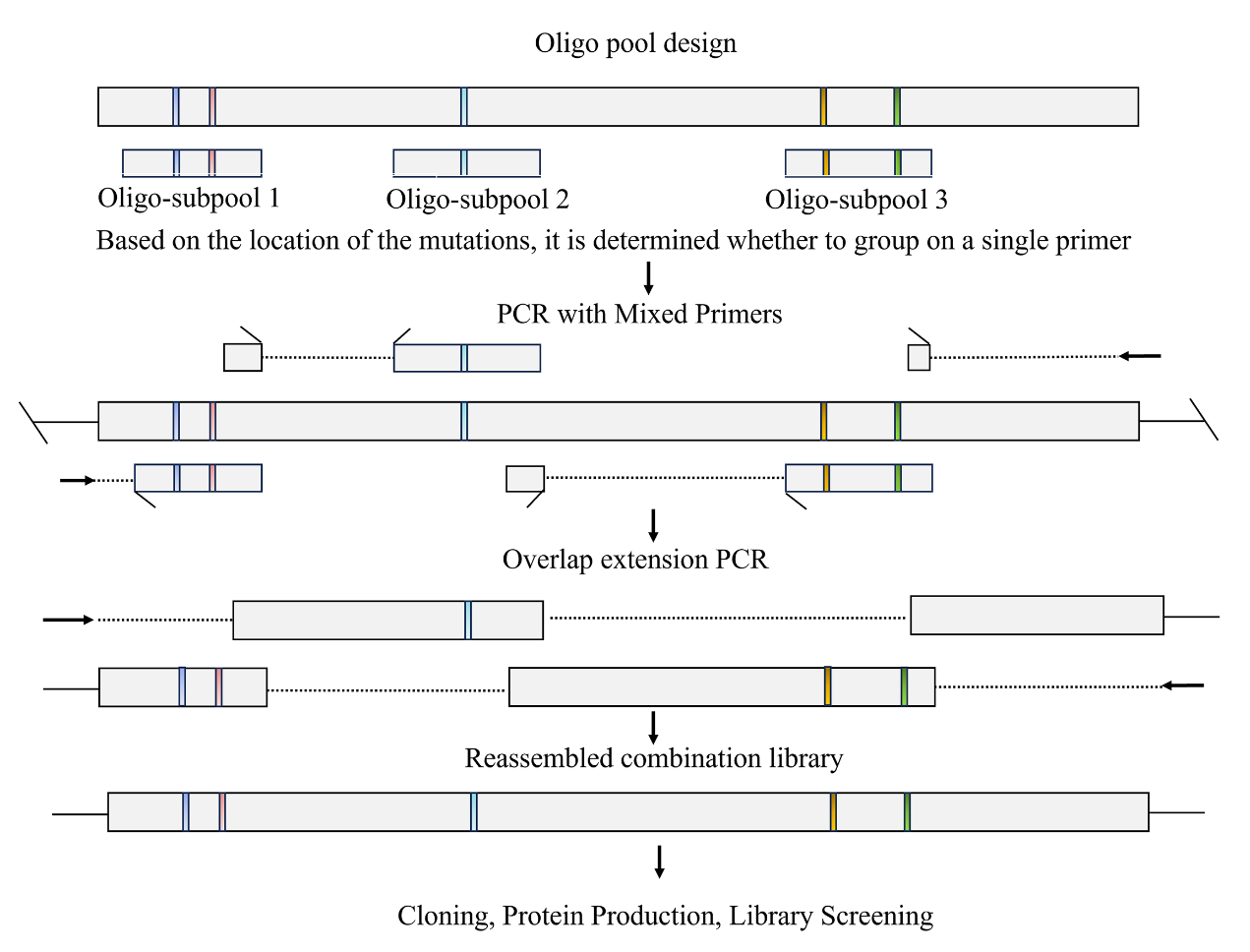


**Fig. S2. Creation of combinatorial libraries.** Mutation sites are first evaluated to determine whether they can be grouped on a single primer; when appropriate, adjacent mutations are combined on one primer. Gene segments from different regions are then amplified using distinct primers. Finally, all mutagenized fragments are assembled via overlap extension PCR to generate the final combinatorial mutant library.


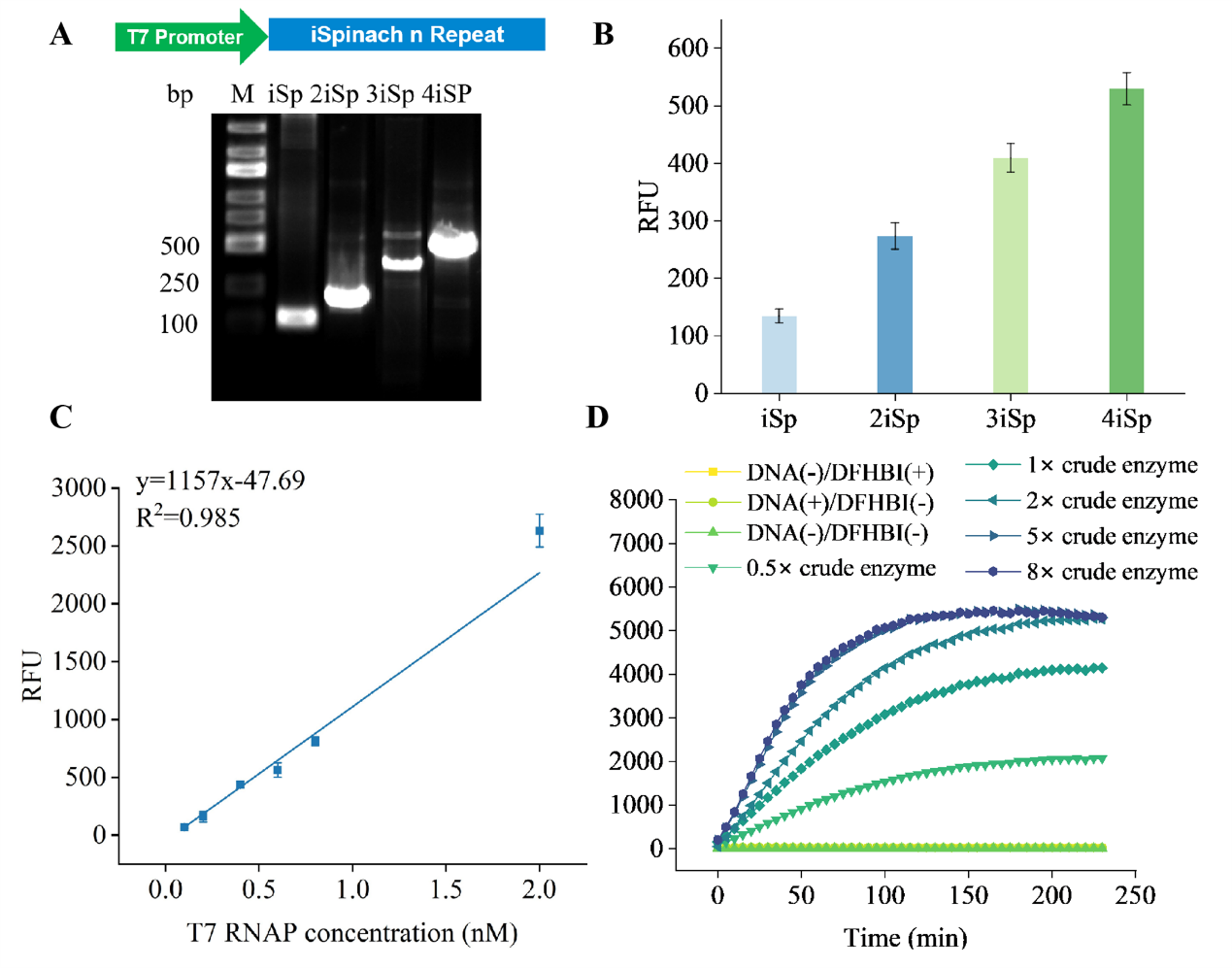


**Fig. S3. Application expansion of the STAR system. (a)** Agarose gel electrophoresis of tandem iSpinach constructs. Labels such as “2–4iSp” indicate the number of tandemly arrayed iSpinach aptamers. **(b)** Relative fluorescence units (RFUs) generated by different tandem iSpinach constructs. Reactions were performed using 50 nM DNA template and 100 nM T7 RNAP, with fluorescence measured after 10 min of transcription. **(c)** RFUs as a function of enzyme concentration, showing a strong linear relationship under constant reaction time. The subfigure shows the excitation and emission spectra of the 4iSp–DFHBI complex. Excitation spectra (blue) were recorded by measuring emission at 550 nm while scanning excitation wavelengths from 380 nm to 500 nm in 2 nm increments. Emission spectra (green) were obtained by exciting the sample at 400 nm and recording fluorescence from 475 nm to 590 nm in 2 nm increments. **(d)** Real-time fluorescence monitoring in crude lysates expressing wild-type T7 RNAP. The T-STAR system remains functional in crude extracts, and fluorescence intensity increases proportionally with lysate concentration.


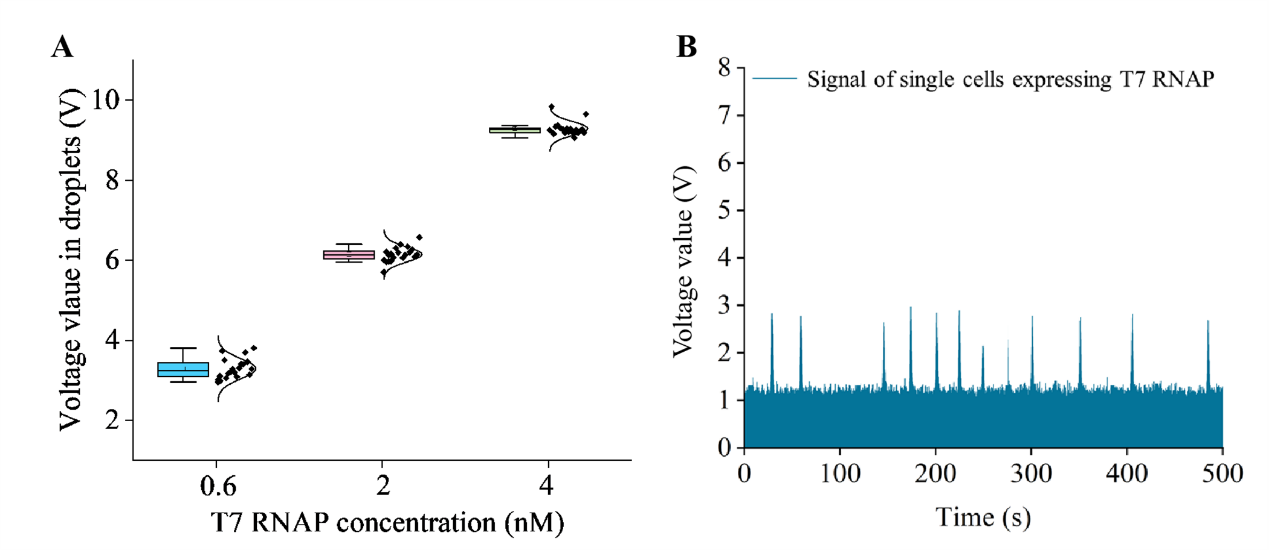


**Fig. S4. Tandem STAR system performs robustly in microdroplets. (A)** Fluorescent signals from droplets containing varying concentrations of T7 RNAP, incubated at 37°C for 20 min. Fluorescence was detected and converted into electrical signals using the FADS system. **(B)** Electrical signal output from microdroplets encapsulating single *E. coli* cells expressing wild-type T7 RNAP after a 2-hour incubation at 37°C. Droplets were subsequently re-injected into the sorting system, and fluorescence-derived signals were recorded.

**
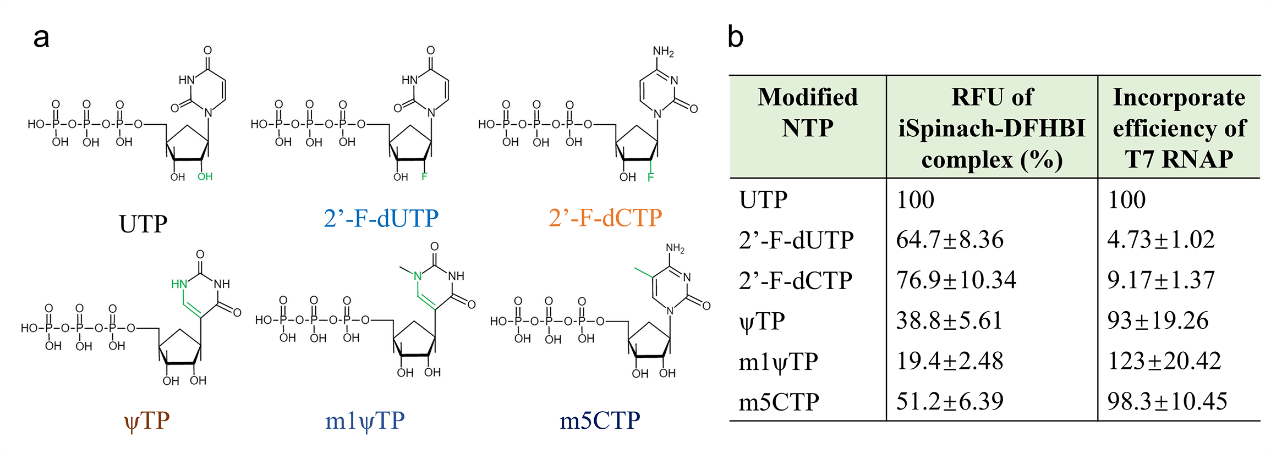
**

**Fig. S5. STAR system compatibility with modified nucleotides.** (a) Chemical structures of the modified nucleotides tested. (b) RFUs of 4iSp aptamers transcribed using various modified nucleotides, reflecting both the fluorescence output and the transcriptional compatibility of T7 RNAP. Although fluorescence intensity was reduced across all modifications, the STAR system effectively transcribed RNAs containing each nucleotide variant.

**
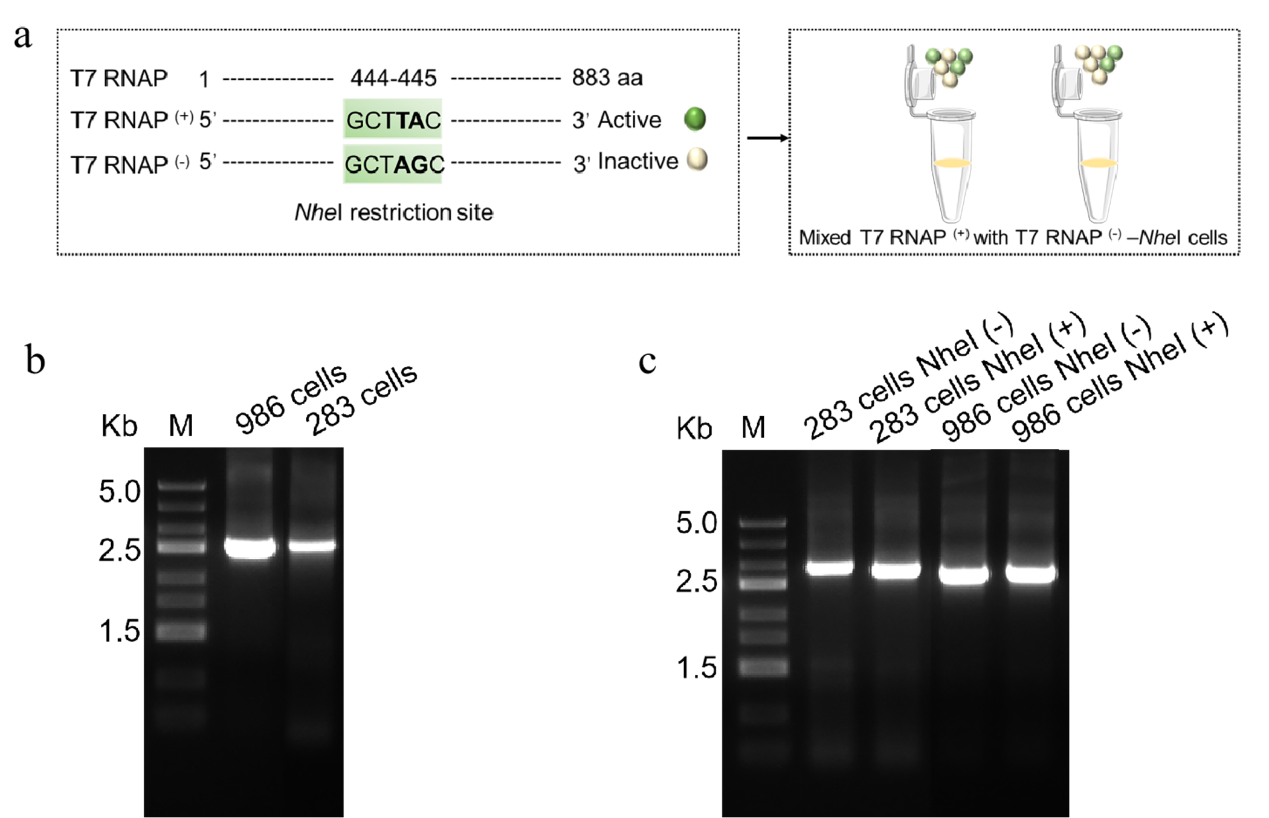
**

**Fig. S6. Evaluation of Ab-FADS screening efficiency. (A)** Schematic of the *Nhe*I restriction site introduced at amino acid positions 444 and 445 of T7 RNAP, generating (i) functional T7 RNAP^(+)^ and (ii) inactive T7 RNAP(–)-*Nhe*I variants. A mixture of both strains was subjected to one round of FADS sorting. **(B)** PCR analysis of sorted droplets, visualized by agarose gel electrophoresis. A total of 986 and 283 positive droplets were collected. **(C)** Recovered genes were digested with *Nhe*I to assess sorting accuracy. Samples labeled “*Nhe*I(–)” were untreated, while “*Nhe*I(+)” samples underwent restriction digestion to determine the fraction of correctly sorted functional clones.


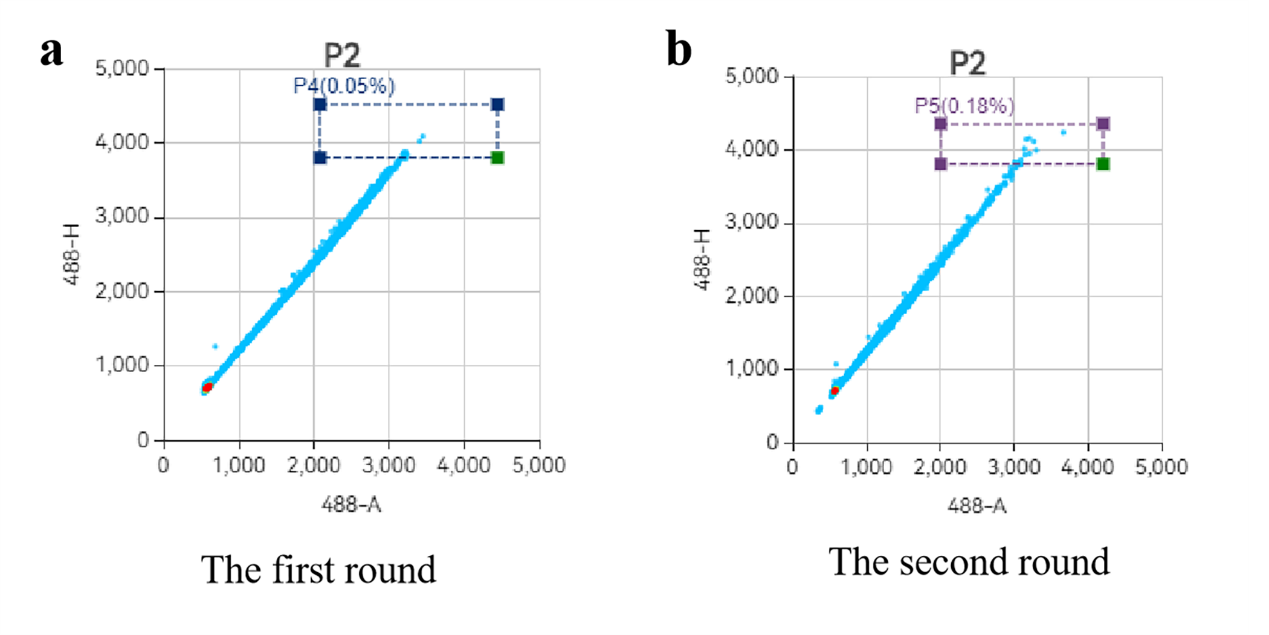


**Fig. S7. Enrichment of the random library using the Ab-FADS platform.** P4 and P5 indicate the positive droplets.

**
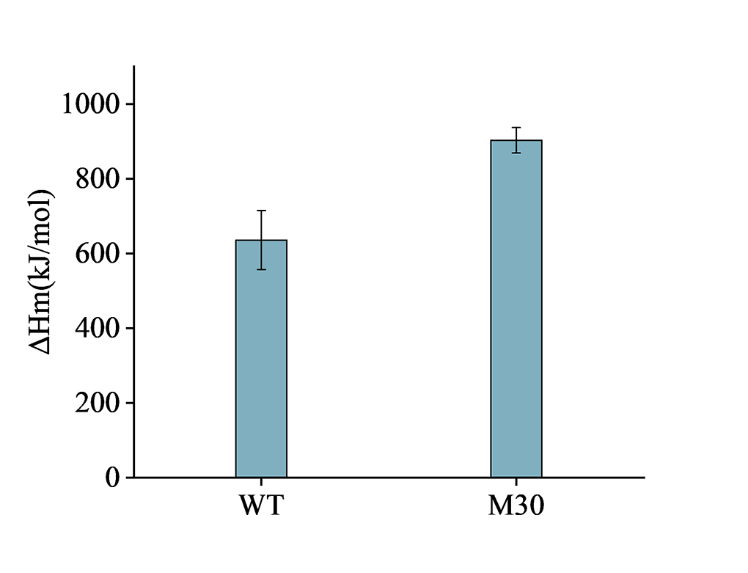
**

**Fig. S8. Thermodynamic analysis of T7 RNAP variants by differential scanning calorimetry (DSC).** Enthalpy change (ΔHm) of wild-type (WT) and M30 mutant T7 RNAP. The M30 mutant exhibits altered thermal stability compared with WT. Values represent the mean ± SD (*n* = 3).


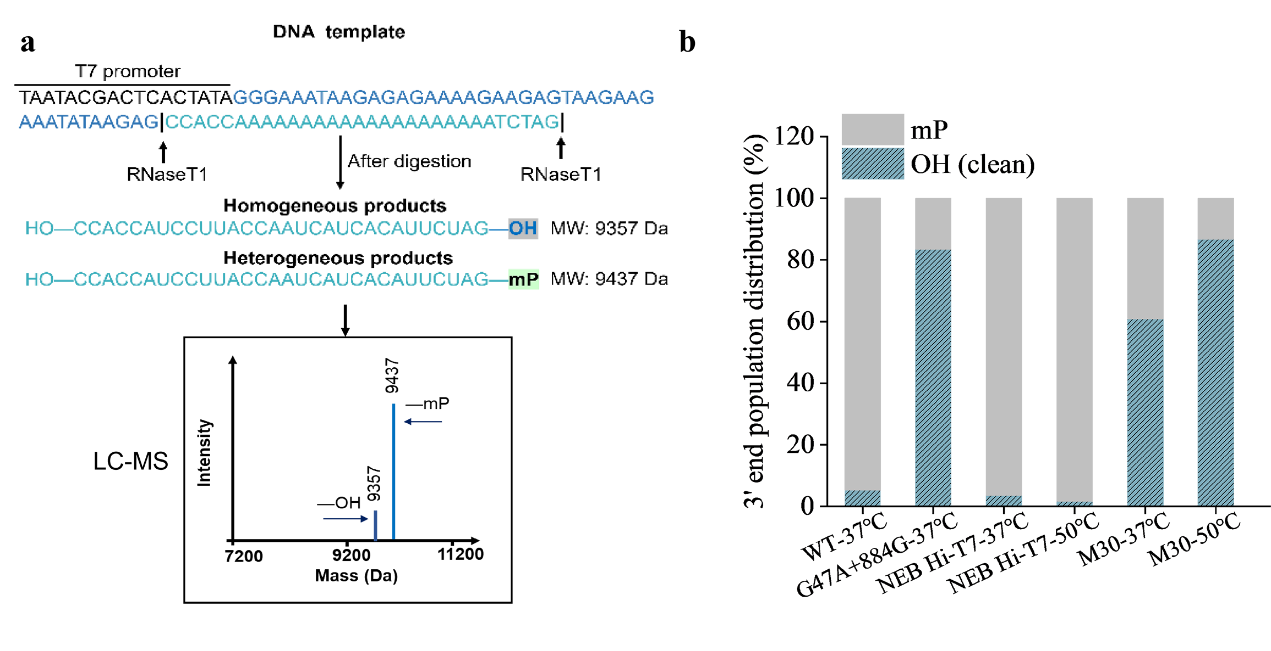


**Fig. S9. Determination of the 3’-end homogeneity of T7 RNAP variants. (a)** Principle for assessing 3′-heterogeneity of transcripts. RNase T1 cleaves mRNA specifically after G nucleotides. Full-length RNA products are not cleaved, leaving a hydroxyl group (–OH), whereas 3′-extended products are cleaved by RNase T1, generating a 3′ monophosphate (mP) “scar.” Thus, RNase T1 digestion can differentiate transcripts with or without non-template additions at the 3′ end. Liquid chromatography–mass spectrometry (LC-MS) is used to determine the ratio of full-length RNA products (n–1, n, n+1) to 3′-extended products based on molecular weight. **(b)** Content of 3′-heterogeneous products and homogeneous IVT products transcribed by different T7 RNAP variants.


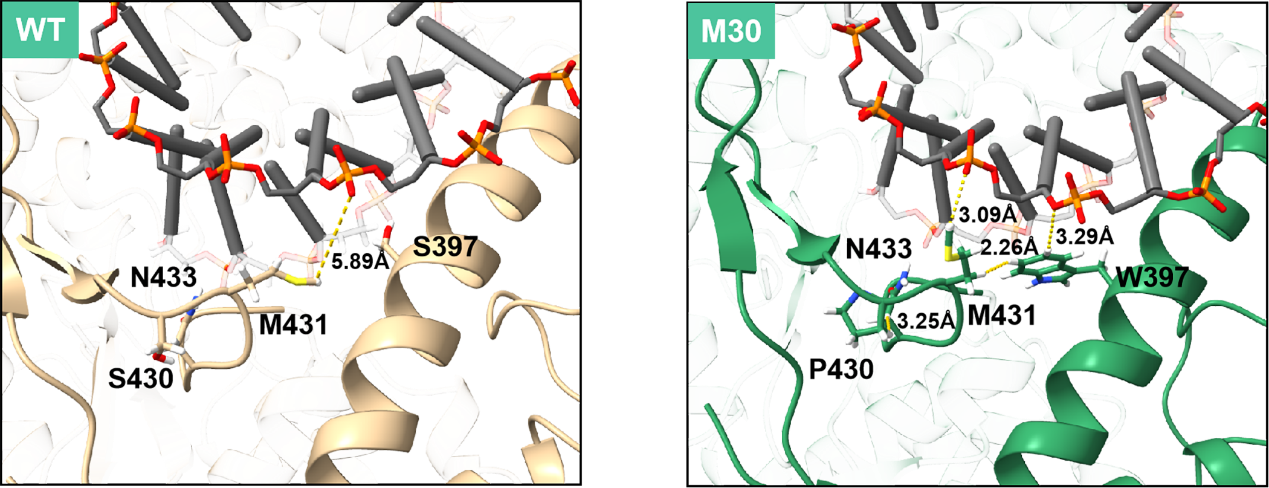


**Fig. S10. Detailed view of residues 397 and 430.** W397 and P430 form increased polar interactions with the surrounding DNA templates.


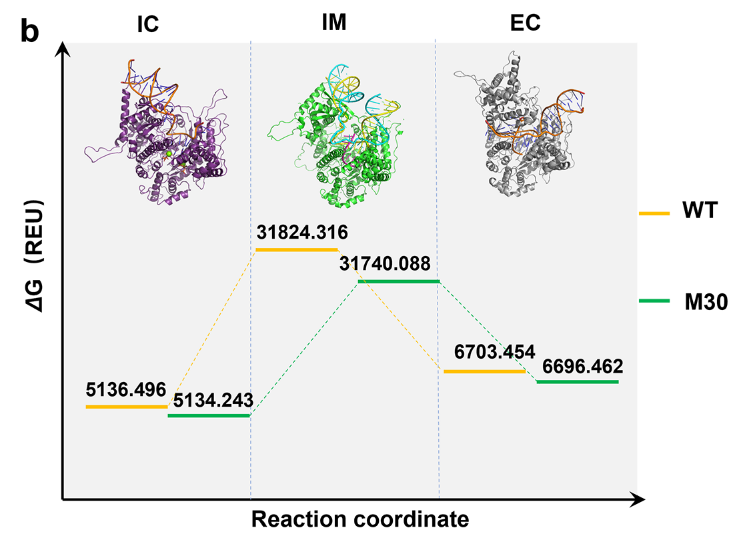


**Fig. S11. Determination of the free energy barrier of T7 RNAP-DNA complexes.** The reaction coordinate axis represents the energy barriers (ΔG, reported in Rosetta Energy Units (REU)) encountered during the conformational transition of T7 RNAP from the initiation complex to the elongation complex.


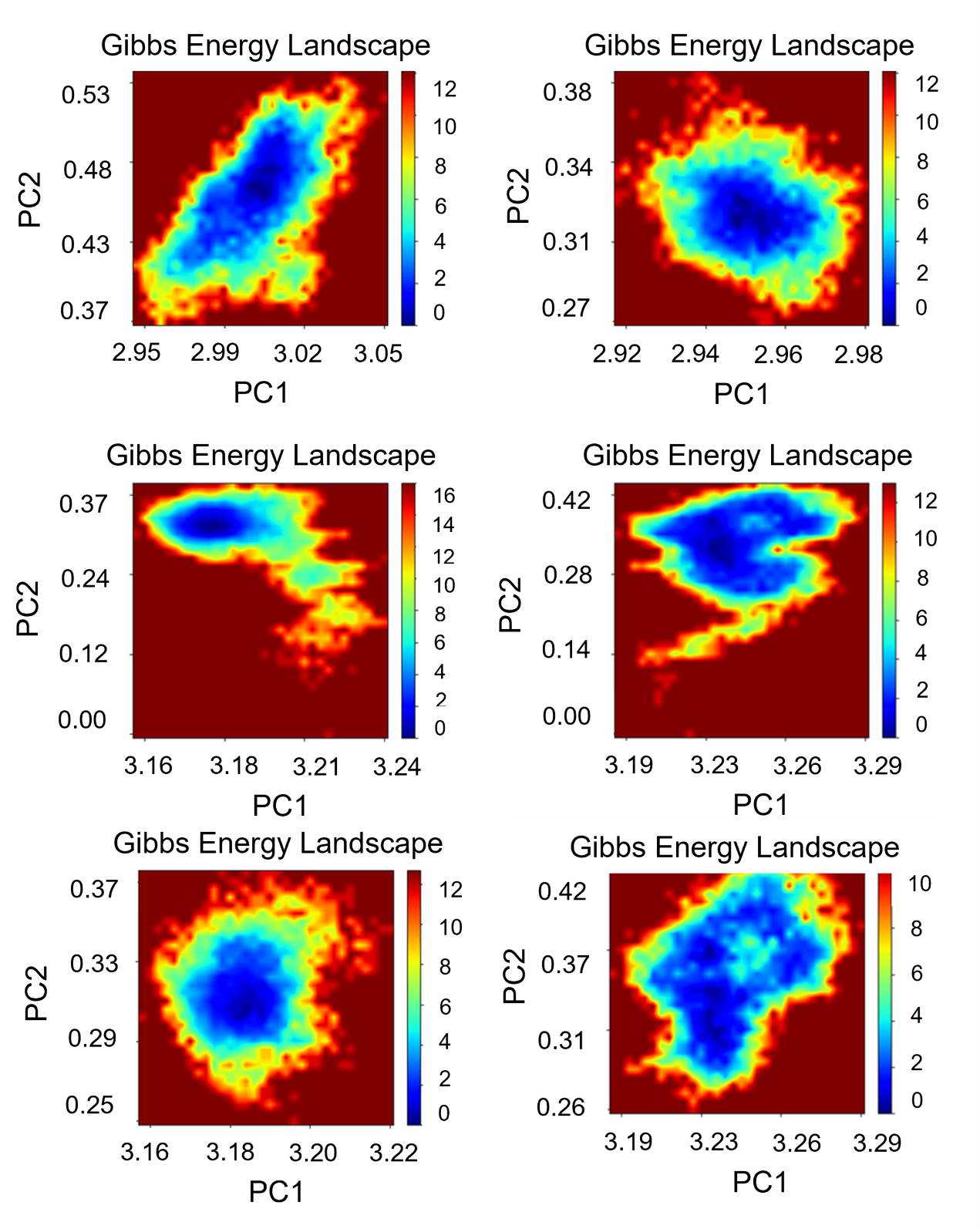


**Fig. S12. Free energy landscape of T7 RNAP in various transcriptional states.** Protein-nucleic acid complex trajectories at steady state were simulated using MD to identify low-energy conformations based on energy minima. The free energy landscape was plotted using root-mean-square deviation (RMSD) and radius of gyration, where principal component 1 (PC1) corresponds to RMSD and principal component 2 (PC2) corresponds to the radius of gyration. Blue indicates lower energy, and red indicates higher energy.

**
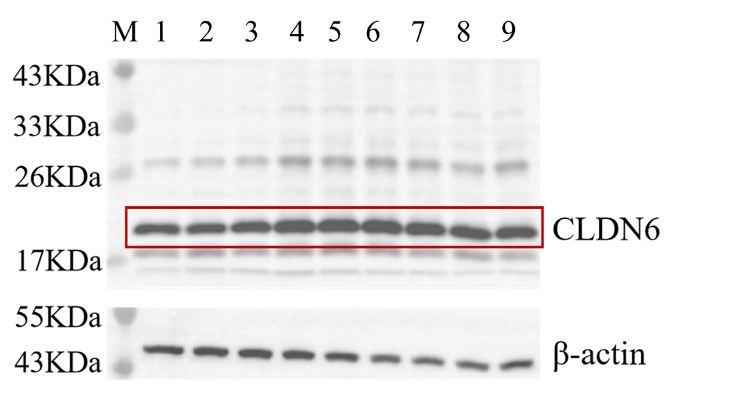
**

**Fig. S13. Western blot analysis of CLDN6 expression driven by WT and M30-derived mRNAs.** CLDN6 protein (theoretical molecular weight: 24 kDa) is highlighted in the red-boxed area; β-actin serves as the internal control. Lane M: molecular weight marker. Lanes 1–3: protein translated from WT-derived mRNA at 37°C; Lanes 4–5: from M30-derived mRNA at 37°C; Lanes 7–9: from M30-derived mRNA at 50°C.


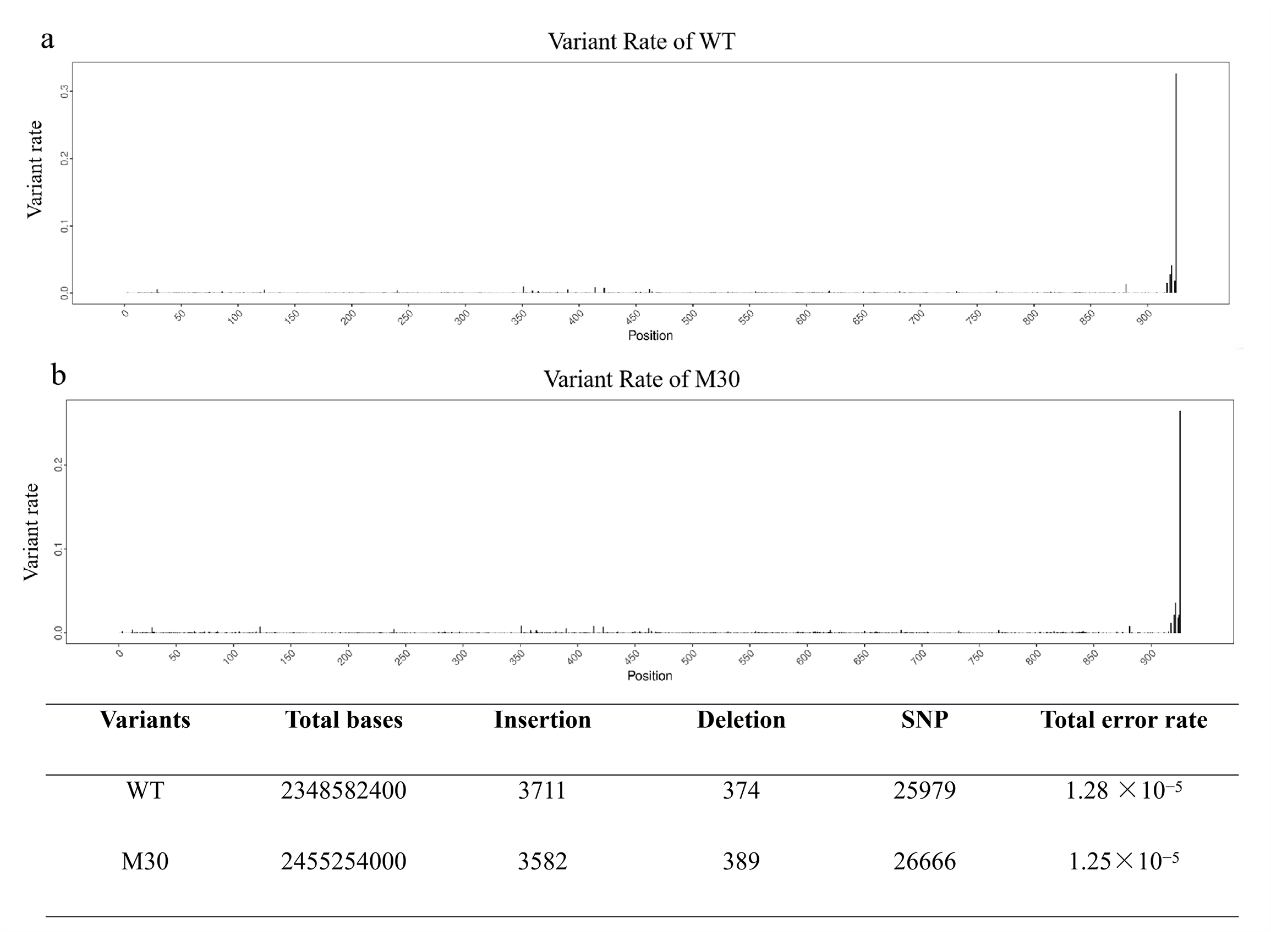


**Fig. S14. Assessment of transcriptional fidelity of T7 RNAP variants by NGS. (a, b)** Variant frequency at each amino acid position of the eGFP mRNA for WT and M30 variants. The table summarizes sequencing statistics, including the total number of bases analyzed, the number of insertion and deletion mutations, single-nucleotide substitutions, and the overall error rate.

1. **Supporting Tables**

**Table S1 T7 RNAP variants obtained using Ab-FADS.**

| Variants | Relative fluorescence units after 50^o^C treatment for 10 min (%) |
| --- | --- |
| WT | 4.60 |
| 1-C3: S397A | 8.28 |
| 1-E5: S397W | 16.10 |
| 1-B8: S397W/S430P | 25.76 |
| M8/V685A | 38.64 |
| M8/Q786L | 59.25 |
| M8/Q786M | 90.16 |
| M8/S633P/Q786M | 153.27 |
| M15/S43L | 245.23 |
| M15/S43R | 183.94 |
| M15/S43Y | 214.57 |
| M15/S43E | 266.80 |

**Table S2 Thermal stability parameters of different T7 RNAP variants.­­**

| Variants | T_m_ (^o^C) | T_50_^15^ (^o^C) | Half-life time at 50^o^C (min) |
| --- | --- | --- | --- |
| WT | 46.50 ± 0.15 | 46.32 ± 0.14 | 0.67 |
| M8 | 51.04 ± 0.24 | 50.56 ± 0.17 | 12.04 |
| M15 | 52.25 ± 0.18 | 52.79 ± 0.19 | 39.15 |
| M25 | 54.52 ± 0.20 | 54.87 ± 0.13 | 90.63 |
| M30 | 55.01 ± 0.21 | 55.01 ± 0.20 | 189.61 |

**Table S3 Primers used in this study.**

| **Name** | **Sequence (5’-3’)** |
| --- | --- |
| pQE-80L-F | AGCGGATAACAATTTCACACAG |
| pQE-80L-R | TTCTGAGGTCATTACTGGATC |
| T7 RNAP -F | GCGGAGCTCATGAACACGATTAACATCGCT |
| T7 RNAP -R | GCGAAGCTTTTACGCGAACGCGAAGT |
| 476X-F | tgtcgataaggttccgttcNNKgagcgcatcaagttcattgaggaaaaccacgagaacatcatggcttgcgctaagtctccactggagaacacttggtgggctgagcaagattctccgttctgcttccttgcgttctgctttgagtacgctggggtacagcaccacggcctgagctataactgctcccttccgctggcgt |
| 476X-R | acgccagcggaaggga |
| 539X-F | tcccttccgctggcgtttgacgggNNKtgctctggcatccagcacttctccgcgatgctccgagatgaggtaggtggtcgcgcggttaacttgcttcctagtgaaaccgttcaggacatctacgggattgttgctaagaaagtcaacgagattctacaagcagacgcaatcaatgggaccgataacgaagtagttaccgt |
| 539X-R | tctcatcggtcacggtaactacttcgtt |
| 633X638X-F | ccgtgaccgatgagaacactggtgaaatctctgagaaagtcaagctgggcactaaggcactggctggtcaatggctggcttacggtgttactcgcagtgtgactaagcgtNNKgtcatgacgctgNNKTACgggtccaaagagttcggcttccgtcaacaagtgctggaagataccattcagccagctattgattccggc |
| 633X638X-R | gaacatcagacccttgccggaatcaata |
| 682X685X-F | caagggtctgatgttcactcagccgaatcaggctgctggatacatggctaagctgattNNKgaatctNNKagcgtgacggtggtagctgcggttgaagcaatgaactggcttaagtctgctgctaagctgctggctgctgaggtcaaagataagaagactggagagattcttcgcaagcgttgcgctgtgcattgggtaa |
| 682X685X-R | aggagttacccaatgcacagc |
| 786X-F | tgcattgggtaactcctgatggtttccctgtgtggcaggaatacaagaagcctattcagacgcgcttgaacctgatgttcctcggtcagttccgcttacagcctaccattaacaccaacaaagatagcgagattgatgcacacaaacaggagtctggtatcgctcctaactttgtacacagcNNKgacggtagccacctt |
| 786X-R | cactacagtcttacgaaggtggctac |
| 36X39X40X43X-F | atgaacacgattaacatcgctaagaacgacttctctgacatcgaactggctgctatcccgttcaacactctggctgaccattacggtgagcgtttagctcgcgaaNNKttggccNNKNNKcatgagNNKtacgagatgggtgaagcacgcttccgcaagatgtttgagcgtcaacttaaagctggtgaggttgcggataa |
| 36X39X40X43X-R | cttggcggcagcgttatccgcaacctcac |
| 59X-F | atgaacacgattaacatcgctaagaacgacttctctgacatcgaactggctgctatcccgttcaacactctggctgaccattacggtgagcgtttagctcgcgaacagttggcccttgagcatgagtcttacgagatgggtgaagcacgcttccgcaagatgtttgagcgtcaaNNKaaagctggtgag |
| 59X-R | aacctcaccagcttt |
| 64X65X67X70X-F | aaagctggtgagNNKNNKgatNNKgctgccNNKaagcctctcatcactaccctactccctaagatgattgcacgcatcaacgactggtttgaggaagtgaaagctaagcgcggcaagcgcccgacagccttccagttcctgcaagaaatcaagccggaagccgtagcgtacatcaccattaagaccactctggcttgcct |
| 64X65X67X70X-R | aggcaagccagagtggtct |
| 810X818X-F | agcctaccattaacaccaacaaagatagcgagattgatgcacacaaacaggagtctggtatcgctcctaactttgtacacagccaagacggtagccaccttcgtaagactgtagtgtgggcacacgagaagtacggaatcgaatcttttgcactgNNKcacgactccttcggtaccattNNKgctgacgctgcgaacctg |
| 810X818X-R | ctttgaacaggttcgcagcgtc |
| 881X-F | acctgttcaaagcagtgcgcgaaactatggttgacacatatgagtcttgtgatgtactggctgatttctacgaccagttcgctgaccagttgcacgagtctcaattggacaaaatgccagcacttccggctaaaggtaacttgaacctccgtgacatcttagagtcggacttcgcgNNKgcgtaaaagctt |
| 881X-R | aagcttttacgc |

**Table S4 Scale-up IVT experiments using the M30 variant.**

| **Reaction volume** | **Yield (mg/mL)** | **dsRNA content (%)** |
| --- | --- | --- |
| 20 µL | 7.6 | 0.05% |
| 10 mL | 8.0 | 0.07% |
| 17.5 mL | 7.4 | 0.06% |

1. **Supporting sequence**

**T7 RNAP sequence:**

atgaacacgattaacatcgctaagaacgacttctctgacatcgaactggctgctatcccgttcaacactctggctgaccattacggtgagcgtttagctcgcgaacagttggcccttgagcatgagtcttacgagatgggtgaagcacgcttccgcaagatgtttgagcgtcaacttaaagctggtgaggttgcggataacgctgccgccaagcctctcatcactaccctactccctaagatgattgcacgcatcaacgactggtttgaggaagtgaaagctaagcgcggcaagcgcccgacagccttccagttcctgcaagaaatcaagccggaagccgtagcgtacatcaccattaagaccactctggcttgcctaaccagtgctgacaatacaaccgttcaggctgtagcaagcgcaatcggtcgggccattgaggacgaggctcgcttcggtcgtatccgtgaccttgaagctaagcacttcaagaaaaacgttgaggaacaactcaacaagcgcgtagggcacgtctacaagaaagcatttatgcaagttgtcgaggctgacatgctctctaagggtctactcggtggcgaggcgtggtcttcgtggcataaggaagactctattcatgtaggagtacgctgcatcgagatgctcattgagtcaaccggaatggttagcttacaccgccaaaatgctggcgtagtaggtcaagactctgagactatcgaactcgcacctgaatacgctgaggctatcgcaacccgtgcaggtgcgctggctggcatctctccgatgttccaaccttgcgtagttcctcctaagccgtggactggcattactggtggtggctattgggctaacggtcgtcgtcctctggcgctggtgcgtactcacagtaagaaagcactgatgcgctacgaagacgtttacatgcctgaggtgtacaaagcgattaacattgcgcaaaacaccgcatggaaaatcaacaagaaagtcctagcggtcgccaacgtaatcaccaagtggaagcattgtccggtcgaggacatccctgcgattgagcgtgaagaactcccgatgaaaccggaagacatcgacatgaatcctgaggctctcaccgcgtgaaacgtgctgccgctgctgtgtaccgcaaggacaaggctcgcaagtctcgccgtatcagccttgagttcatgcttgagcaagccaataagtttgctaaccataaggccatctggttcccttacaacatggactggcgcggtcgtgtttacgctgtgtcaatgttcaacccgcaaggtaacgatatgaccaaaggactgcttacgctggcgaaaggtaaaccaatcggtaaggaaggttactactggctgaaaatccacggtgcaaactgtgcgggtgtcgataaggttccgttccctgagcgcatcaagttcattgaggaaaaccacgagaacatcatggcttgcgctaagtctccactggagaacacttggtgggctgagcaagattctccgttctgcttccttgcgttctgctttgagtacgctggggtacagcaccacggcctgagctataactgctcccttccgctggcgtttgacgggtcttgctctggcatccagcacttctccgcgatgctccgagatgaggtaggtggtcgcgcggttaacttgcttcctagtgaaaccgttcaggacatctacgggattgttgctaagaaagtcaacgagattctacaagcagacgcaatcaatgggaccgataacgaagtagttaccgtgaccgatgagaacactggtgaaatctctgagaaagtcaagctgggcactaaggcactggctggtcaatggctggcttacggtgttactcgcagtgtgactaagcgttcagtcatgacgctggcttacgggtccaaagagttcggcttccgtcaacaagtgctggaagataccattcagccagctattgattccggcaagggtctgatgttcactcagccgaatcaggctgctggatacatggctaagctgatttgggaatctgtgagcgtgacggtggtagctgcggttgaagcaatgaactggcttaagtctgctgctaagctgctggctgctgaggtcaaagataagaagactggagagattcttcgcaagcgttgcgctgtgcattgggtaactcctgatggtttccctgtgtggcaggaatacaagaagcctattcagacgcgcttgaacctgatgttcctcggtcagttccgcttacagcctaccattaacaccaacaaagatagcgagattgatgcacacaaacaggagtctggtatcgctcctaactttgtacacagccaagacggtagccaccttcgtaagactgtagtgtgggcacacgagaagtacggaatcgaatcttttgcactgattcacgactccttcggtaccattccggctgacgctgcgaacctgttcaaagcagtgcgcgaaactatggttgacacatatgagtcttgtgatgtactggctgatttctacgaccagttcgctgaccagttgcacgagtctcaattggacaaaatgccagcacttccggctaaaggtaacttgaacctccgtgacatcttagagtcggacttcgcgttcgcgtaa

**iSpinach sequence：**

GTCAGATCCATAATACGACTCACTATAGGGGCGACTACGGTGAGGGTCGGGTCCAGTAGCTTCGGCTACTGTTGAGTAGAGTGTGGGCTCCGTAGTCGC

**2iSpinach sequence:**

GTCAGATCCATAATACGACTCACTATAGGGGCGACTACGGTGAGGGTCGGGTCCAGTAGCTTCGGCTACTGTTGAGTAGAGTGTGGGCTCCGTAGTCGCacgtaagatgctccggttagggaGCGACTACGGTGAGGGTCGGGTCCAGTAGCTTCGGCTACTGTTGAGTAGAGTGTGGGCTCCGTAGTCGC

**3iSpinach sequence:**

GAATTCGTCAGATCCATAATACGACTCACTATAGGGGCGACTACGGTGAGGGTCGGGTCCAGTAGCTTCGGCTACTGTTGAGTAGAGTGTGGGCTCCGTAGTCGCacgtaagatgctccggttagggaGCGACTACGGTGAGGGTCGGGTCCAGTAGCTTCGGCTACTGTTGAGTAGAGTGTGGGCTCCGTAGTCGCacgtaagatgctccggttagggaGCGACTACGGTGAGGGTCGGGTCCAGTAGCTTCGGCTACTGTTGAGTAGAGTGTGGGCTCCGTAGTCGCacgtaagatgctccggttagggaGGATCC

**4iSpinach sequence:**

GAATTCGTCAGATCCATAATACGACTCACTATAGGGGCGACTACGGTGAGGGTCGGGTCCAGTAGCTTCGGCTACTGTTGAGTAGAGTGTGGGCTCCGTAGTCGCacgtaagatgctccggttagggaGCGACTACGGTGAGGGTCGGGTCCAGTAGCTTCGGCTACTGTTGAGTAGAGTGTGGGCTCCGTAGTCGCacgtaagatgctccggttagggaGCGACTACGGTGAGGGTCGGGTCCAGTAGCTTCGGCTACTGTTGAGTAGAGTGTGGGCTCCGTAGTCGCacgtaagatgctccggttagggaGCGACTACGGTGAGGGTCGGGTCCAGTAGCTTCGGCTACTGTTGAGTAGAGTGTGGGCTCCGTAGTCGCacgtaagatgctccggttagggaGGATCC

**Template for specific activity determination of T7 RNAP**

TTATCGAAATTAATACGACTCACTATAGGGAGACCCAAGCTGGCTAGCCTTGTTCTTTTTGCAGAAGCTCAGAATAAACGCTCAACTTTGGGCCACCATGGACGCCATGAAGAGAGGCCTGTGCTGCGTGCTGCTCCTGTGCGGCGCCGTGTTCGTGAGCGCTAGAGTGCAGCCCACCGAGAGCATCGTGAGATTCCCCAACATCACCAACCTGTGCCCCTTCGGCGAGGTGTTCAACGCCACAAGATTCGCCTCCGTGTACGCCTGGAATAGAAAGAGAATCAGCAACTGCGTGGCCGACTACAGCGTGCTGTACAACAGCGCCTCCTTCAGCACATTTAAGTGCTACGGCGTGAGCCCCACCAAGCTGAACGACCTGTGCTTCACCAACGTGTACGCCGACAGCTTCGTGATCAGAGGCGACGAGGTGAGACAGATCGCCCCCGGGCAGACCGGCAAGATCGCCGACTACAACTACAAGCTGCCCGACGACTTCACCGGCTGCGTGATCGCCTGGAACAGCAACAACCTGGACAGCAAGGTGGGCGGCAACTACAACTACCTGTACAGACTGTTCAGAAAGAGCAACCTGAAGCCCTTCGAGAGAGACATCAGCACCGAGATCTACCAAGCCGGCAGCACCCCCTGCAACGGCGTGGAGGGCTTCAACTGCTACTTCCCCCTGCAGAGCTACGGCTTTCAGCCCACCAACGGCGTGGGCTATCAGCCCTACAGAGTGGTCGTGCTGAGCTTCGAGCTGCTGCACGCCCCCGCCACCGTGTGCGGCCCCAAGAAGAGCACCAACCTGGTGAAGAACAAGTGCGTCAACTTTCATCATCACCATCACCACTGAAACCAGCCTCAAGAACACCCGAATGGAGTCTCTAAGCTACATAATACCAACTTACACTTTACAAAATGTTGTCCCCCAAAATGTAGCCATTCGTATCTGCTCCTAATAAAAAGAAAGTTTCTTCACTCTAGAGGGCCCGTTTAAACCCGCTGATCAGCCTCGACTGTGCCTTCTAG

**Template for 3’-homogeneity determination and radioactive-gel-based analysis of IVT products**

TAATACGACTCACTATAGGGAAATAAGAGAGAAAAGAAGAGTAAGAAGAAATATAAGAGCCACCAAAAAAAAAAAAAAAAAAAATCTAG

**mEPO sequence:**

aagtgccacctgaccggtcgataatacgactcactataAggAAATAAGAGAGAAAAGAAGAGTAAGAAGAAATATAAGAGCCACCATGGGAGTGCCCGAAAGACCTACACTGCTCCTGCTGCTGTCTCTGCTGCTGATCCCTCTGGGACTGCCTGTGCTTTGTGCCCCTCCTAGACTGATCTGCGACAGCAGAGTGCTGGAACGGTACATCCTGGAAGCCAAAGAGGCCGAGAACGTCACCATGGGCTGTGCTGAAGGCCCAAGACTGAGCGAGAACATCACCGTGCCTGACACCAAAGTGAACTTCTACGCCTGGAAGAGGATGGAAGTGGAAGAACAGGCCATCGAAGTGTGGCAGGGACTGAGCCTGCTGTCTGAGGCTATTCTGCAGGCACAGGCCCTGCTGGCCAACTCTTCTCAGCCTCCTGAAACACTGCAGCTCCACATCGACAAGGCCATCAGCGGCCTGAGAAGCCTGACCTCTCTGCTGAGAGTTCTGGGCGCTCAGAAAGAACTGATGAGCCCTCCTGACACAACCCCTCCAGCTCCTCTGAGAACTCTGACCGTGGACACCTTCTGCAAGCTGTTCAGAGTGTACGCCAACTTCCTGAGAGGCAAGCTGAAGCTGTACACCGGCGAAGTGTGCAGACGGGGCGATAGATAAGCGGCCGCTTAATTAAGCTGCCTTCTGCGGGGCTTGCCTTCTGGCCATGCCCTTCTTCTCTCCCTTGCACCTGTACCTCTTGGTCTTTGAATAAAGCCTGAGTAGGAAGAAAAAAAAAAAAAAAAAAAAAAAAAAAAAAGCATATGACTAAAAAAAAAAAAAAAAAAAAAAAAAAAAAAAAAAAAAAAAAAAAAAAAAAAAAAAAAAAAAAAAAAAAAATGAGACCTgaagagc

**CLDN6 sequence:**

aagtgccacctgaccggtcgataatacgactcactataaggGAGAATAAACTAGTATTCTTCTGGTCCCCACAGACTCAGAGAGAACCCGCCACCATGGCCTCTGCCGGAATGCAGATCCTGGGCGTGGTGCTGACCCTGCTGGGCTGGGTGAATGGCCTGGTGAGCTGTGCCCTGCCCATGTGGAAGGTGACAGCCTTCATTGGCAACAGCATTGTGGTGGCCCAGGTGGTGTGGGAGGGCCTGTGGATGAGCTGTGTGGTGCAGAGCACAGGCCAGATGCAGTGCAAGGTGTATGACAGCCTGCTGGCCCTGCCTCAGGACCTCCAGGCCGCCAGAGCCCTGTGTGTGATTGCCCTGCTGGTGGCCCTGTTTGGCCTGCTGGTGTACCTGGCTGGAGCCAAGTGCACCACCTGTGTGGAGGAGAAGGACAGCAAGGCCAGACTGGTGCTGACCTCTGGCATTGTGTTTGTGATCTCTGGCGTGCTGACCCTGATCCCTGTGTGCTGGACAGCCCATGCCATCATCAGAGACTTCTACAACCCTCTGGTGGCCGAGGCCCAGAAAAGAGAGCTGGGAGCCAGCCTGTACCTGGGCTGGGCCGCCTCTGGCCTTCTTCTGCTGGGAGGAGGACTGCTGTGCTGCACCTGCCCCTCTGGCGGCAGCCAGGGCCCCAGCCACTACATGGCCAGATACAGCACCTCTGCCCCTGCCATCAGCAGAGGCCCTTCTGAGTACCCCACCAAGAACTATGTGGGAGGATCCGGTGGTGGCGGCAGCGGCGGCTGACTCGAGCTGGTACTGCATGCACGCAATGCTAGCTGCCCCTTTCCCGTCCTGGGTACCCCGAGTCTCCCCCGACCTCGGGTCCCAGGTATGCTCCCACCTCCACCTGCCCCACTCACCACCTCTGCTAGTTCCAGACACCTCCCAAGCACGCAGCAATGCAGCTCAAAACGCTTAGCCTAGCCACACCCCCACGGGAAACAGCAGTGATTAACCTTTAGCAATAAACGAAAGTTTAACTAAGCTATACTAACCCCAGGGTTGGTCAATTTCGTGCCAGCCACACCGAGACCTGGTCCAGAGTCGCTAGCCGCGTCGCTAAAAAAAAAAAAAAAAAAAAAAAAAAAAAAGCATATGACTAAAAAAAAAAAAAAAAAAAAAAAAAAAAAAAAAAAAAAAAAAAAAAAAAAAAAAAAAAAAAAAAAAAAAATgaagagc

**eGFP sequence：**

aagtgccacctgaccggtcgataatacgactcactatagggaaataagagagaaaagaagagtaagaagaaatataagaccccggcgccgccaccatggtgagcaagggcgaggagctgttcaccggggtggtgcccatcctggtcgagctggacggcgacgtaaacggccacaagttcagcgtgtccggcgagggcgagggcgatgccacctacggcaagctgaccctgaagttcatctgcaccaccggcaagctgcccgtgccctggcccaccctcgtgaccaccctgacctacggcgtgcagtgcttcagccgctaccccgaccacatgaagcagcacgacttcttcaagtccgccatgcccgaaggctacgtccaggagcgcaccatcttcttcaaggacgacggcaactacaagacccgcgccgaggtgaagttcgagggcgacaccctggtgaaccgcatcgagctgaagggcatcgacttcaaggaggacggcaacatcctggggcacaagctggagtacaactacaacagccacaacgtctatatcatggccgacaagcagaagaacggcatcaaggtgaacttcaagatccgccacaacatcgaggacggcagcgtgcagctcgccgaccactaccagcagaacacccccatcggcgacggccccgtgctgctgcccgacaaccactacctgagcacccagtccgccctgagcaaagaccccaacgagaagcgcgatcacatggtcctgctggagttcgtgaccgccgccgggatcactctcggcatggacgagctgtacaagtaagctggagcctcggtggcctagcttcttgccccttgggcctccccccagcccctcctccccttcctgcacccgtacccccgtggtctttgaataaagtctgagtgggcggcAAAAAAAAAAAAAAAAAAAAAAAAAAAAAAGCATATGACTAAAAAAAAAAAAAAAAAAAAAAAAAAAAAAAAAAAAAAAAAAAAAAAAAAAAAAAAAAAAAAAAAAAAAATgaagagc

**DNA template used for determination of binding Affinity:**

5’-6-FAM-AAAATAATACGACTCACTATAGGGAGACCCTCGAGGACAGATCAAAAAGATCTGTCCTCGAGGGTCTCCCTATAGTGAGTCGTATTATTTT

1. **Supporting materials and methods**

**4.1 Fluorescent microscopy**

Droplets were mixed with HFE 7500 oil and applied to glass slides for microscopic imaging. Bright-field and fluorescence images were acquired using 20× and 40× objectives on a LEICA DM2000 microscope (Leica Microsystems CMS GmbH). For single-cell encapsulation analysis, at least 100 droplets were examined under bright-field illumination to ensure statistical reliability. Fluorescence was detected in the same field of view under dark-field conditions using an FITC filter set.

**4.2 Detection of fluorescence signals in microdroplets**

To evaluate fluorescence output at varying concentrations of purified T7 RNAP, a 2× T-STAR reaction system containing 0, 0.6, 2, or 4 nM T7 RNAP was co-encapsulated with a 2× T-STAR solution lacking T7 RNAP using a droplet generation device. The resulting droplets were incubated at 37°C for 20 min, followed by heat inactivation at 65°C for 10 min. Droplets were then re-injected into a detection/sorting chip to quantify fluorescence signal intensity based on voltage output for each T7 RNAP concentration.

To detect fluorescence signals from single cells expressing WT T7 RNAP, the previously described droplet-based library screening method was employed. For model validation, two droplet types were prepared: positive droplets containing cells expressing WT T7 RNAP and negative droplets containing cells harboring an *Nhe*I-inactivated T7 RNAP construct. These droplets were mixed at ratios of 1:1 and 1:2, and the top 0.5% of highly fluorescent droplets were isolated using the detection/sorting chip.

Sorted droplets were disrupted using a demulsifier to release the encapsulated cells. A 50 μL PCR reaction was set up containing 25 μL 2× PrimerSTAR Max, 2 μL each of pQE-80L-F and pQE-80L-R primers (10 μM), and ddH_2_O. The recovered gene products were digested with *Nhe* I for 30 min to verify the presence or absence of the restriction site. Agarose gel electrophoresis was used to compare band patterns before and after digestion. Since WT T7 RNAP lacks the *Nhe* I site, while the inactivated construct contains it, unchanged band sizes after digestion confirmed successful recovery of WT positive clones.

**4.3 Molecular dynamics simulations**

Molecular dynamics (MD) simulations were performed on a DNA/RNA–T7 RNAP complex in a periodic box, maintaining a minimum distance of 1 nm between the solute and the box boundary. The system was solvated with TIP3P water molecules and neutralized with 0.1 mol/L NaCl. The CHARMM27 force field was applied for protein simulations. All MD simulations were conducted using GROMACS 2023 (1).

Van der Waals interactions were truncated at 1.2 nm using a Lennard-Jones potential, with a smooth taper to zero starting at 1.0 nm. Electrostatic interactions were calculated using the particle mesh Ewald method with a Coulomb cutoff of 1.2 nm. Bond constraints involving hydrogen atoms were applied using the LINCS algorithm, allowing a time step of 2 fs.

Energy minimization was performed using the steepest descent method for up to 50,000 steps or until the maximum force dropped below 10.0 kJ·mol⁻^1^·nm⁻^1^. Equilibration was conducted in the NVT ensemble at 335.15 K for 50 ns, followed by NPT ensemble equilibration at 1 bar for an additional 50 ns. Temperature was controlled using the velocity-rescale thermostat (τ = 0.1 ps), and pressure was maintained with the Parrinello-Rahman barostat (τ = 3 ps). Production MD simulations were performed in the NPT ensemble for 200 ns, with snapshots recorded every 100 ps. The final 100 ns of trajectories were used for analysis. A representative, the key GROMACS input parameter file (grompp.mdp) used for the production run is provided in https://github.com/sci-master/t7rnap.git.

Hydrogen bonds between T7 RNAP and DNA were analyzed over the entire production trajectory using the GROMACS gmx hbond tool. A hydrogen bond was defined geometrically with a donor–acceptor distance cutoff of 3.5 Å and a hydrogen–donor–acceptor angle cutoff of 30°. The mean number of hydrogen bonds at each frame throughout the equilibrium trajectory was computed. Analysis was performed after confirming system equilibration, verified by the convergence of the root-mean-square deviation (RMSD) of the protein and DNA backbone atoms. To ensure statistical robustness, the trajectory was divided into three independent blocks, and the average hydrogen bond number was calculated for each block; the reported value represents the mean ± standard deviation across these blocks. The occupancy of each specific intermolecular hydrogen bond was calculated as the percentage of simulation time during which the bond existed relative to the total equilibrium simulation time. Hydrogen bonds with occupancy greater than 10% were considered significant. The script used for this hydrogen bond analysis (gmx_mmpbsa.bsh) is provided in https://github.com/sci-master/t7rnap.git for reference.

To construct the free energy landscape (FEL), MD trajectories were preprocessed using the trjconv tool in GROMACS to remove translational and rotational motions. RMSD and radius of gyration (Rg) were calculated using rms and gyrate, and the combined trajectory data were input into the *sham* utility to generate FEL maps in XPM format. Protein structures were visualized using PyMOL (2).

**4.4 Characterization of the relative and specific activity of T7 RNAP variants**

His-tagged T7 RNAP was expressed in Escherichia coli BL21(DE3) carrying the pQE-80L plasmid and purified as previously described(24). Protein concentration was determined using the Residual T7 RNA Polymerase Detection Kit (Hzyme). The purified T7 RNAP was diluted to a final concentration of 5 mg/mL in storage buffer (50 mM Tris–HCl, pH 8.0, 100 mM NaCl, 100 μM EDTA-Na⁺, 1 mM dithiothreitol, 75% glycerol), aliquoted, and stored at −80°C.

The relative activities of T7 RNAP variants were measured using the T-STAR system. For iSpinach aptamer DNA templates with varying tandem repeats, reactions contained 50 nM DNA and 100 nM T7 RNAP, with other components unchanged, and were incubated at 37°C for 20 min, followed by endpoint fluorescence measurement. Temperature-dependent activity assays were conducted using the same T-STAR system, with reactions performed at temperatures ranging from 25°C to 55°C for 20 min. For crude lysate activity determination, 0.5–8 µL (0.5–8×) of high-pressure homogenized lysate was monitored via real-time fluorescence detection using the T-STAR system. All experiments were performed in triplicate. True fluorescence values were obtained by subtracting the control (no T7 RNAP) signal, and data were processed using Origin 9.

The specific activities of the enzymes were quantified using a pyrophosphate-coupled spectrophotometric assay. During in vitro transcription, each nucleotide incorporation releases inorganic pyrophosphate (PPi), which is hydrolyzed by inorganic pyrophosphatase (PPase) to generate phosphate (Pi). Pi is then measured through the purine nucleoside phosphorylase (PNPase)-catalyzed conversion of 7-methyl-6-thioguanosine (MESG) into 7-methyl-6-thioguanine (MTGua), with the absorbance of MTGua directly reflecting PPi production and, consequently, polymerase activity. The reaction principle is as follows:

The 300 µL transcription reaction contained 1× transcription buffer, 1 mM NTPs, 0.1 kU/mL PNPase (Yuanye Bio-Technology), 0.2 mM MESG (Solarbio), 0.001 U/µL PPase (Thermo Fisher Scientific), 0.4 kU/mL RNase inhibitor, 80 nM 1 Kb DNA template (Hzymes), and 50 nM T7 RNAP. Enzyme activity was assayed at 37°C or 50°C using a dual-beam spectrophotometer (Thermo Fisher). Absorbance at 360 nm was monitored for 7 min, with linear phase analysis (200–400 s) used to exclude mixing artifacts and substrate depletion. Triplicate measurements were corrected using blank baselines. Specific activity (U/mg) was calculated as:


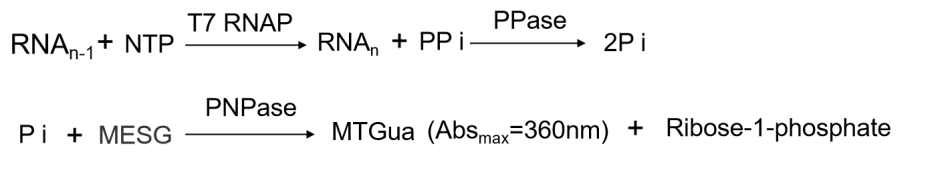


(ΔA: absorbance change; Vtotal: total reaction volume (mL); df: dilution factor; C: enzyme concentration in mg/mL; Ɛ: molar absorptivity of the chromophore at 360 nm (cm^2^/mmol); 1: cuvette pathlength (cm); Venzyme: the volume of T7 RNAP; T: reaction time (h)). One unit of activity is defined as the amount of enzyme required to catalyze the incorporation of 1 nmol PPi into MTGua at 37°C over 1 min.

**4.5 Determination of 3’-homogeneity**

The 3′-end homogeneity of transcription products was evaluated using an RNase T1-based assay (3). RNase T1 specifically cleaves RNA at the 3′ side of guanosine residues. Full-length transcripts terminating with a 3′-hydroxyl (–OH) are resistant to cleavage, whereas 3′-extended products containing misincorporations or overextensions are cleaved, generating 3′-monophosphate (mP) “scars.” Thus, RNase T1 digestion can distinguish transcripts with or without non-template additions at the 3′ end. Liquid chromatography–mass spectrometry (LC-MS) was used to determine the ratio of full-length RNA products (n–1, n, n+1) to 3′-extended products based on their molecular weights.

For the assay, 40 µL of mRNA (1 mg/mL; sequence provided in Supporting Information) was denatured in a solution containing 60 µL of 8 M urea, 12 µL of 1 M Tris-HCl (pH 7.0), and 0.8 µL of 0.5 M EDTA. The mixture was heated to 90°C for 10 min and then cooled to room temperature. Subsequently, 20 µL of RNase T1 (1,000 U/µL) was added, and samples were incubated at 37°C for 15 min. After digestion, RNA fragments were purified and analyzed by reversed-phase ion-pairing liquid chromatography using an Agilent 1290 UPLC system coupled to an Agilent 6530 Q-TOF mass spectrometer.

**4.6 Thermostability assays**

To evaluate the thermostability of T7 RNAP variants, purified enzymes were diluted to 1 mg/mL in storage buffer (50 mM Tris-HCl, 100 mM NaCl, 100 μM EDTA, 2 mM DTT, 75% glycerol). Enzyme half-life (T_1/2_) was determined by incubating the enzyme at 50°C for varying durations, followed by immediate cooling and initiation of transcription reactions at 37°C. Residual activity was measured, and T_1/2_ was calculated using the equation T_1/2_ = −ln(2)/k, where *k* is the slope from the linear portion of the plot of the natural logarithm of relative residual activity versus heating time.

To determine T_50_^15^ values, enzymes in storage buffer were incubated at temperatures ranging from 45°C to 60°C for 15 min, followed by rapid cooling on ice. Transcriptional activity was then measured at 37°C, with activity at this temperature defined as 100%. The T_50_^15^ value— defined as the temperature at which 50% of activity remains after a 15-minute incubation—was calculated by fitting the data to a sigmoidal Boltzmann curve using Origin 9.0. All thermostability assays were performed in triplicate, and mean values were reported for each variant.

**4.7 Differential scanning calorimetry**

Thermal stability was further assessed using a nano differential scanning calorimetry (DSC) system (TA Instruments, USA). T7 RNAP variants were diluted to 1 mg/mL in PBS (pH 7.5), with PBS alone serving as the reference. All samples were degassed at 4°C for 15 min to remove air bubbles. DSC scans were performed under 3 bar pressure from 5°C to 95°C at a heating rate of 1°C/min, following a 600-second equilibration. The baseline signal (reference–reference) was subtracted from the sample–reference scan. Thermal unfolding profiles were analyzed using a two-state scaled model.

**4.8 Radioactive-gel-based analysis of IVT products**

IVT reactions (20 µL) were assembled using 20 nM WT or M30 mutant enzyme, 10 mM each of ATP, UTP, GTP, and CTP, 0.5 µCi/µL α-³²P-GTP or α-³²P-CTP, 1.8 μM dsDNA template, 1 μL RNase inhibitor (20 U/μL), and 100 U/mL pyrophosphatase in 1× IVT buffer A. The DNA template was generated by annealing synthetic oligonucleotides containing a T7 promoter (same sequences as used in the 3′-homogeneity assay). Reactions were incubated for 1 hour at 37°C or 50°C and stopped with 80 mM EDTA. RNA products were purified via LiCl precipitation and resuspended in 1× RNA loading dye. Samples were denatured at 75°C for 2 min and separated on a 20% denaturing acrylamide gel (6 M urea), initially run at 20 W for 30 min and then at 40 W for 2 hours. Gels were exposed to phosphor screens and imaged with a Typhoon FLA9500 Biomolecular Imager. For densitometric analysis, autoradiographs were processed in ImageJ. For each lane, fixed rectangular regions of interest (ROIs) were defined to correspond to full-length RNA, longer RNA species (including loopback dsRNA and 3’-extended products), and truncated RNA species. Background signal was determined from adjacent signal-free regions within the same lane and subtracted. Signal intensities of individual RNA species were calculated as fractions of the total RNA signal in the corresponding lane (full-length + longer + truncated RNA). Relative byproduct content was obtained by normalizing these fractions to the corresponding RNA species under the WT-37^o^C condition, which was defined as 1.

**4.9 Western blot**

THP-1 cells were seeded in 12-well plates at 1.5 × 10^5^ cells per well and cultured overnight. Cells were transfected with mRNA using Lipofectamine™ MessengerMAX (LMRNA015, Invitrogen, USA) following the manufacturer’s protocol. Forty-eight hours post-transfection, cell lysates were collected and separated by 10% SDS-PAGE, then transferred to 0.45 μm PVDF membranes (Millipore, USA). Membranes were blocked with 5% non-fat milk and incubated with anti-CLDN6 antibody (1:1000, A24011, ABclonal, China), followed by HRP-conjugated secondary antibody (1:5000, SA00001-2, Proteintech, China). β-Actin (1:5000, 66009-1-Ig, Proteintech, China) was used as a loading control.

**4.10 Transcription fidelity of T7 RNAP**

The fidelity of T7 RNA polymerase was assessed using an eGFP reporter template. Linearized DNA containing a T7 promoter was transcribed in vitro under standard conditions (37°C, 2 h). After DNase I treatment, RNA products were purified and reverse-transcribed using a high-fidelity reverse transcriptase. The resulting cDNA was submitted to Genewiz (Suzhou, China) for library preparation and Illumina paired-end sequencing. Briefly, cDNA fragments were end-repaired, A-tailed, and ligated to Illumina adapters with unique indexes. Libraries were quality-controlled and sequenced following standard protocols. The DNA template was sequenced in parallel as a control to correct for pre-existing mutations and sequencing errors. Sequencing reads were quality-filtered, adapter-trimmed, and mapped to the eGFP reference, and mutation frequencies were calculated to determine the overall transcription fidelity.

**References**

1. Páll, S., Zhmurov, A., Bauer, P., Abraham, M., Lundborg, M., Gray, A., Hess, B. and Lindahl, E. (2020) Heterogeneous parallelization and acceleration of molecular dynamics simulations in GROMACS. J Chem Phys, 153.

2. Schrödinger, L., & DeLano, W. (2020). PyMOL. Retrieved from <http://www.pymol.org/pymol.>

3. Jiang, T., Yu, N., Kim, J., Murgo, J.-R., Kissai, M., Ravichandran, K., Miracco, E.J., Presnyak, V. and Hua, S. (2019) Oligonucleotide Sequence Mapping of Large Therapeutic mRNAs via Parallel Ribonuclease Digestions and LC-MS/MS. Analytical Chemistry, 91, 8500-8506.
